# Diversity of Pharmaceuticals Enhances Antibiotic Resistance in the Invertebrate Gut via Biofilm‐Mediated Mechanisms

**DOI:** 10.1002/advs.202518849

**Published:** 2026-04-13

**Authors:** Yi‐Fei Wang, Ya‐Ning Wang, Da Lin, Jia‐Yang Xu, Feng‐Yuan Qi, Hui‐Ling Cui, Hui‐Jie Lu, Min Qiao, Edward Topp, Dong Zhu, Matthias C. Rillig, Yong‐Guan Zhu

**Affiliations:** ^1^ State Key Laboratory of Regional and Urban Ecology Ningbo Observation and Research Station Institute of Urban Environment Chinese Academy of Sciences Xiamen China; ^2^ Zhejiang Key Laboratory of Pollution Control for Port‐Petrochemical Industry CAS Haixi Industrial Technology Innovation Center in Beilun Ningbo China; ^3^ University of Chinese Academy of Sciences Beijing China; ^4^ State Key Laboratory of Regional and Urban Ecology Research Center for Eco‐Environmental Sciences Chinese Academy of Sciences Beijing China; ^5^ Key Laboratory of Environment Remediation and Ecological Health Ministry of Education College of Environmental Resource Sciences Zhejiang University Hangzhou China; ^6^ London Research and Development Centre (LRDC) Agriculture and Agri‐Food Canada London Canada; ^7^ Institute of Biology Freie Universität Berlin Berlin Germany; ^8^ Berlin‐Brandenburg Institute of Advanced Biodiversity Research (BBIB) Berlin Germany

**Keywords:** antibiotic resistance genes, biofilm, pharmaceutical diversity, soil invertebrate, warming

## Abstract

The environmental accumulation of non‐antibiotic pharmaceuticals is an emerging driver of antibiotic resistance. While individual compounds are known to shape the soil resistome, and contaminant diversity also plays a role, the impact of pharmaceutical diversity on the gut resistome of soil invertebrates remains unclear. Here, we combined metagenomics and metaproteomics to examine the collembolan gut and soil resistome across a gradient of pharmaceutical diversity under diurnal warming. Increasing pharmaceutical diversity at a constant total concentration significantly enriched antibiotic resistance genes (ARGs) in the gut microbiome, with no comparable effect in surrounding soils. This enrichment was mainly driven by multidrug resistance associated with efflux activity and biofilm‐related processes, accompanied by increases in ARG‐carrying taxa such as *Gordonia* and *Ochrobactrum*. Notably, *Ochrobactrum* encoded biofilm‐related aryl polyene pathways. In vitro experiments confirmed that biofilm formation promotes resistance through coordinated cellular responses. Metaproteomic data indicated that *Ochrobactrum* initiates early biofilm formation by recruiting extracellular matrix producers such as *Bacillus* and *Pseudomonas*. Diurnal warming modulated these responses, indicating an interaction between chemical diversity and climate stress. These findings identify pharmaceutical diversity as an independent driver of ARG enrichment in host‐associated microbiomes and establish chemical complexity as a key factor in assessing the ecological risks of pharmaceutical pollution.

## Graphical abstract

Pharmaceutical diversity acts as an independent driver of antibiotic resistance in soil invertebrates. While bulk soil remains unaffected, the collembolan gut microbiome exhibits significant resistance gene enrichment under complex chemical exposure and diurnal warming. This adaptation is driven by chemotaxis‐mediated biofilm formation, highlighting the critical role of chemical complexity in global antimicrobial resistance surveillance.

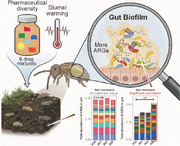

## 1 Introduction

The rapid rise in antibiotic resistance represents a critical global health challenge, threatening the efficacy of existing antimicrobial therapies and complicating the treatment of infectious diseases [1]. The environmental dissemination of antibiotic resistance genes (ARGs) is a key driver of this crisis, enabling resistance to persist and spread beyond clinical settings [2]. Soils are fundamental to agricultural productivity, ecosystem functioning, and represent major reservoirs of ARGs [3]. Soil invertebrates, including earthworms and collembolans, regulate nutrient cycling and organic matter turnover [4], while also acting as ecological hubs for ARG accumulation and trophic transfer [5, 6]. Within the One Health framework, resolving ARG dynamics in soil‐animal systems is therefore essential for understanding how environmental reservoirs contribute to the broader resistance burden [7].

Non‐antibiotic pharmaceuticals comprise diverse chemical classes widely used in human and veterinary medicine and are ubiquitously released into the environment [8, 9]. These compounds have been detected globally across various environmental matrices [10, 11, 12, 13]. Soils act as critical environmental sinks that continuously receive inputs from organic fertilizers, sewage sludge, and contaminated irrigation water, resulting in the frequent co‐occurrence of multiple pharmaceuticals as complex mixtures [11]. Crucially, a significant proportion of these non‐antibiotic compounds can inhibit bacterial growth, highlighting their potential to promote bacterial resistance alongside traditional antibiotics [14]. Recent advancements in environmental resistome research have begun to address the risks of chemical mixtures. For instance, studies have demonstrated that simple binary or ternary combinations of pollutants can synergistically enrich ARGs in soils [15, 16], and the diversity of emerging contaminants, such as microplastics, has been newly identified as a driver of soil ARG proliferation [17]. However, research on non‐antibiotic pharmaceuticals remains largely confined to the dose‐dependent effects of single compounds [18]. Consequently, a critical knowledge gap remains regarding the specific ecological impact of pharmaceutical diversity, which refers to the number of distinct chemical compounds acting simultaneously at environmentally relevant concentrations. Furthermore, soil invertebrates are directly exposed to these complex chemical mixtures through ingestion [19, 20]. Because the gut microbiome of soil invertebrates is often more sensitive to pollutants than bulk soil microbial communities [21], we hypothesized that pharmaceutical diversity may drive antibiotic resistance primarily within host‐associated microbiome rather than in bulk soils.

The mechanisms by which non‐antibiotic substances contribute to antibiotic resistance are complex. Individual compounds can promote ARG enrichment via distinct mechanisms, including enhanced horizontal gene transfer through elevated reactive oxygen species (ROS) and stimulation of efflux pump activity [22, 23]. Studies have shown that multiple co‐occurring pollutants, such as pesticide mixtures, significantly amplify microbial stress responses [24]. Similarly, exposure to highly diverse pharmaceutical mixtures likely induces severe, compounded oxidative stress. To survive such harsh multi‐chemical environments, microbial communities often aggregate to form biofilms as a collective defense strategy [25]. Within these dense biofilm structures, the close physical proximity of cells and elevated stress levels synergistically accelerate horizontal gene transfer, making them recognized hotspots for ARG dissemination [26, 27, 28]. However, the mechanism of ARG response and the specific role of biofilm formation in response to varying levels of pharmaceutical diversity remain unclear. Given that the gut lumen of invertebrates provides an ideal, confined niche for biofilm colonization, we hypothesized that increasing pharmaceutical diversity may enrich the gut resistome by driving biofilm‐mediated mechanisms.

Climate warming has emerged as a major driver of antibiotic resistance [29]. Sustained increases in mean temperature are known to enhance horizontal gene transfer of ARGs and alter the environmental fate of pollutants [30, 31, 32]. Yet, most studies have focused on constant warming, despite the fact that global climate change also amplifies the frequency and magnitude of daily temperature fluctuations [33]. Soil ectotherms such as collembolans are highly sensitive to microclimatic variation, and diurnal temperature shifts often impose greater physiological stress than predictable, constant warming [34, 35]. Such stress can induce gut microbial oxidative damage and disrupt homeostasis [36], potentially modulating the effects of co‐occurring chemical contaminants. However, the effects of realistic diurnal warming on soil‐animal resistome remain largely unexplored.

To investigate the effects and underlying mechanisms of pharmaceutical diversity on ARGs in soil‐animal systems, we exposed collembolans to combinations of up to seven non‐antibiotic pharmaceuticals frequently co‐detected in terrestrial environments [11]. In parallel, virulence factor genes (VFGs) were analyzed as complementary indicators of bacterial competitiveness to provide a broader assessment of ecological risks. To realistically simulate climate warming scenarios, we implemented a diurnal temperature fluctuation treatment (+3°C over a 24 h cycle relative to a standard 20°C baseline), an increment chosen to represent the projected global mean temperature rise by the end of the 21st century [37]. Specifically, we (1) quantified ARG responses in soil and collembolan guts across a pharmaceutical diversity gradient, (2) resolved differences between soil and gut resistome, (3) compared gut ARG responses under constant and fluctuating temperature regimes, and (4) elucidated the mechanisms linking pharmaceutical diversity to ARG enrichment. Our findings demonstrate that chemical diversity, beyond pollutant identity or concentration, acts as an independent driver of ARG dissemination, particularly within host‐associated microbiomes under realistic warming scenarios.

## 2 Results

### 2.1 Increasing Diversity of Pharmaceuticals Drives ARG and VFG Dynamics in Collembolan Guts

**FIGURE 1:**
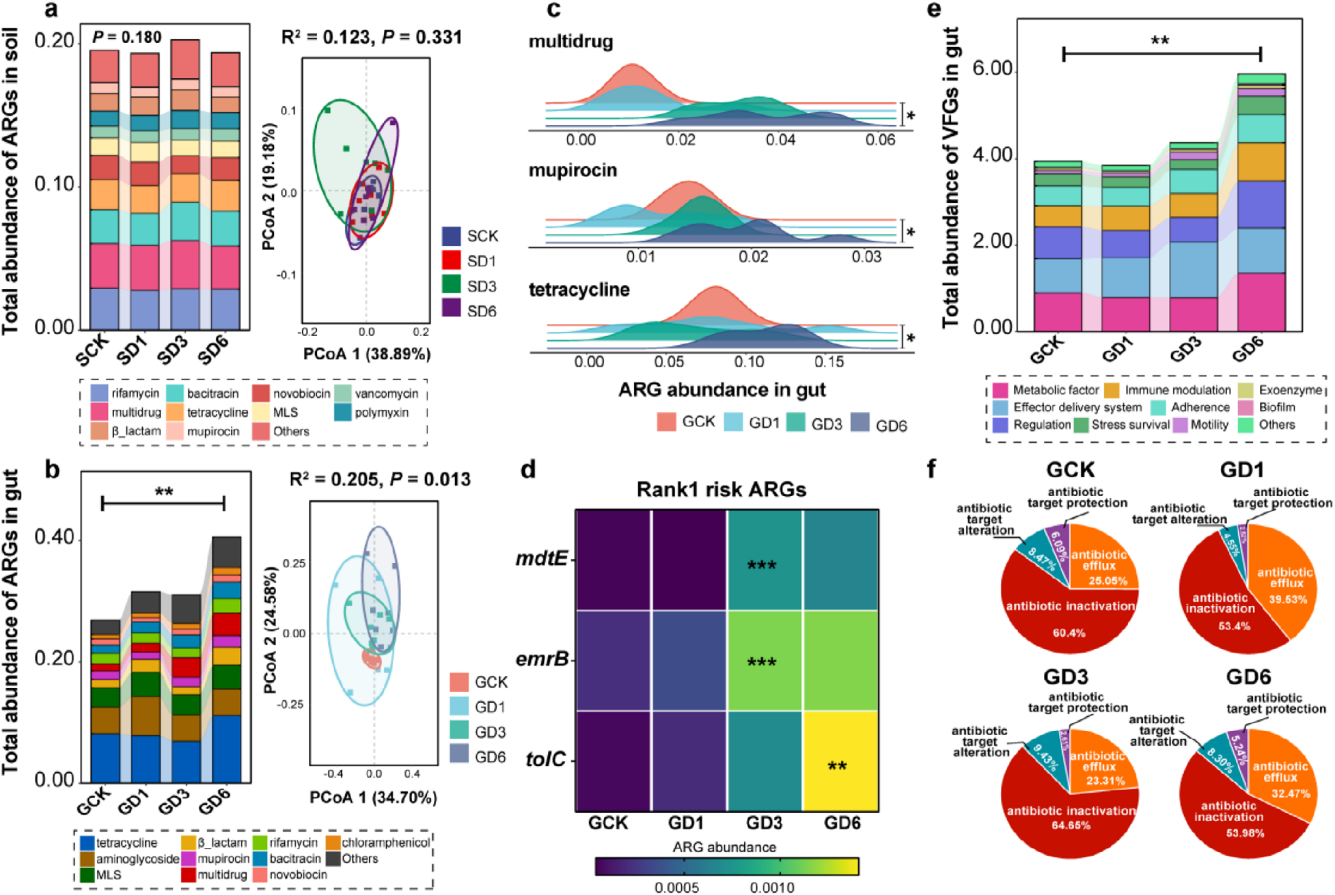
Increasing diversity of pharmaceuticals modulates ARG and VFG profiles in soil and the collembolan gut. (a) Stacked bar plot (left) showing the total relative abundance of ARGs in soil under increasing pharmaceutical diversity treatments (SCK: control; SD1, SD3, SD6: 1, 3, and 6 drugs, respectively; *n* = *7* per group). Principal coordinates analysis (PCoA, right) based on Bray–Curtis distances reveals no significant compositional shifts in soil ARGs. (b) Total relative abundance of ARGs in collembolan guts significantly increased with pharmaceutical diversity (GCK: control; GD1, GD3, GD6: 1, 3, and 6 drugs, respectively; *n* = 7 per group, except GD1 where *n* = 6 due to one sample failing amplification), with clear compositional separation revealed by PCoA. (c) Density plots illustrate the relative abundance distribution of representative ARG types (multidrug, mupirocin, and tetracycline) in gut samples under increasing pharmaceutical diversity (*n* = 7 per group, GD1 *n* = 6). (d) Heatmap showing relative abundance of high‐risk ARGs across gut samples (*n* = 7 per group, GD1 *n* = 6). (e) Total relative abundance of VFGs in collembolan guts increased significantly with pharmaceutical diversity (*n* = 7 per group, GD1 n = 6). (f) Pie charts depicting the relative contributions of ARG functional categories in each treatment group. Data are presented as means. Differences among treatments were evaluated using the Kruskal–Wallis test. Significance among treatments is denoted as *p < 0.05, **p < 0.01, ***p < 0.001. The unit of genes is copies per cell.

Across all samples, we identified 742 ARG subtypes in soil and 524 in the collembolan gut, spanning 27 resistance categories. Soil ARGs were dominated by those conferring resistance to rifamycin (14.75%) and multidrug antibiotics (14.79%), whereas aminoglycosides (30.32%) and tetracycline (17.70%) resistance genes predominated in the collembolan gut. Pharmaceutical diversity did not significantly influence the total abundance (*p* = 0.180, Kruskal–Wallis test) or compositional profile (*p* = 0.331, PERMANOVA test) of soil ARGs (Figure 1a). In contrast, high pharmaceutical diversity significantly elevated total ARG abundance (GD6 vs. GCK: *p* = 0.007, Kruskal–Wallis test) and altered ARG community composition in collembolan guts (*p* = 0.013, PERMANOVA test, Figure 1b). Notably, multidrug (*p* = 0.019, Kruskal–Wallis test), mupirocin (*p* = 0.042, Kruskal–Wallis test), and tetracycline (*p* = 0.050, Kruskal–Wallis test) resistance genes were significantly enriched when collembolans were exposed to six types of pharmaceuticals (Figure 1c).

Rank 1 high‐risk ARGs, including *mdtE* (GD3 vs. GCK: *p* < 0.001, Kruskal–Wallis test), *emrB* (GD3 vs. GCK: *p* < 0.001, Kruskal–Wallis test), and *tolC* (GD6 vs. GCK: *p* = 0.004, Kruskal–Wallis test), all associated with multidrug resistance, were significantly enriched with increasing pharmaceutical diversity in the collembolan gut (Figure 1d). Concurrently, VFGs were also significantly elevated (GD6 vs. GCK: *p* = 0.002, Kruskal–Wallis test, Figure 1e), particularly those involved in antimicrobial activity (GD3 vs. GCK: *p* < 0.001, Kruskal–Wallis test), biofilm formation (GD6 vs. GCK: *p* = 0.004, Kruskal–Wallis test), and stress survival (GD6 vs. GCK: *p* = 0.005, Kruskal–Wallis test) (Figure S1). In terms of mechanisms of antimicrobial resistance, pharmaceutical exposure increased the relative contribution of ARGs related to antibiotic efflux pumps in collembolan gut (Figure 1f).

### 2.2 Increasing Diversity of Pharmaceuticals Enriches ARG‐VFG‐MGE Co‐Carriers in Collembolan Guts

**FIGURE 2:**
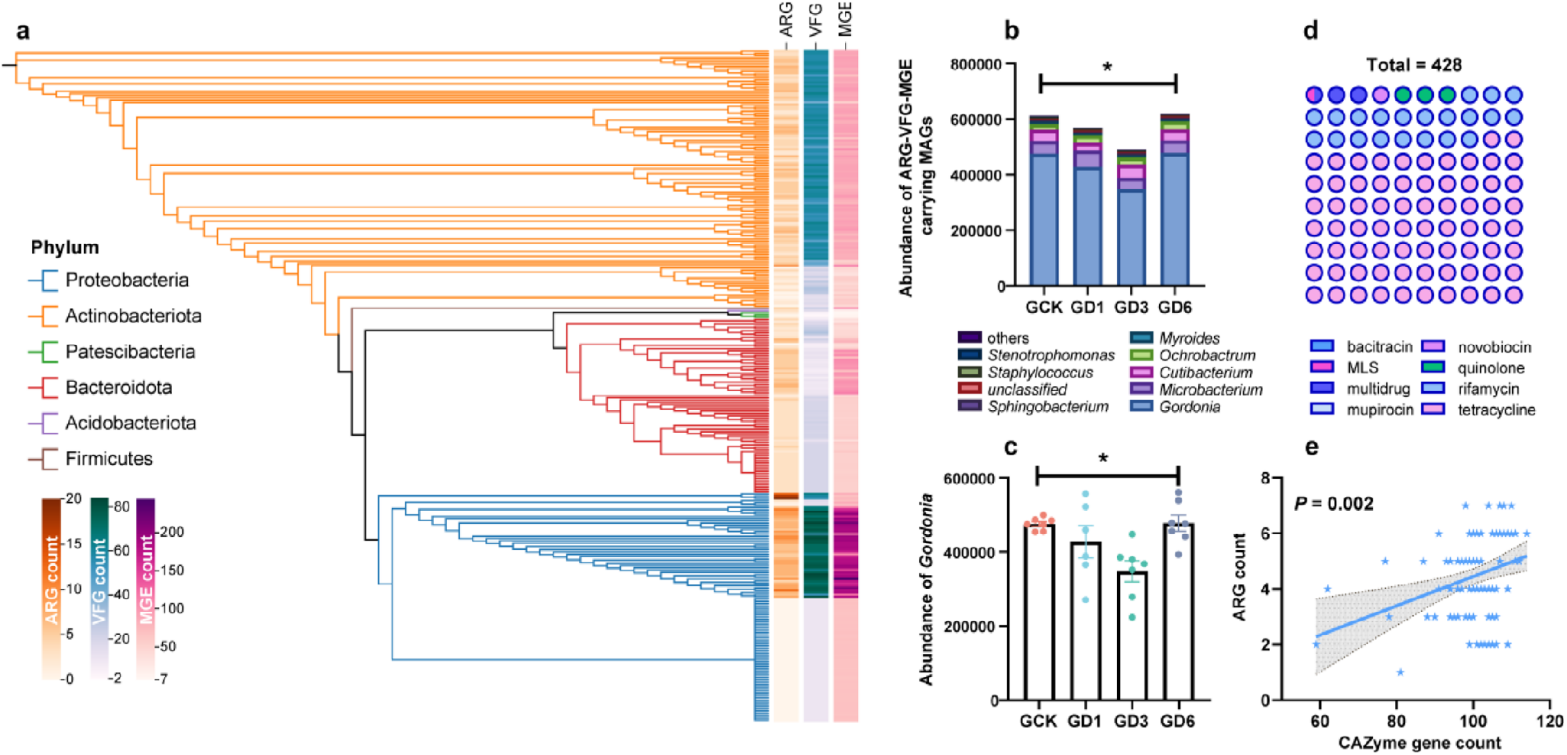
Increasing diversity of pharmaceuticals drives the expansion of ARG–VFG–MGE co‐carriers in collembolan gut. (a) Phylogenetic tree of representative metagenome‐assembled genomes (MAGs), annotated with the presence of ARGs, VFGs, and MGEs. (b) Stacked bar plots showing the increasing abundance of ARG–VFG–MGE co‐carriers in collembolan guts across pharmaceutical diversity treatments (GCK: control; GD1, GD3, GD6: 1, 3, and 6 drugs, respectively; *n* = 7 per group, except GD1 where *n* = 6 due to one sample failing amplification). (c) Histogram illustrating the rise in *Gordonia* abundance in collembolan guts with increasing pharmaceutical diversity (*n* = 7 per group, GD1 *n* = 6). (d) Proportional composition of ARG categories encoded by *Gordonia* across all gut MAGs. (e) Correlation between CAZyme and ARG gene counts in *Gordonia*. Shaded bands represent 95% confidence intervals from linear regression. Error bars represent mean ± standard error (SE). Differences among treatments were evaluated using the Kruskal–Wallis test. Significance among treatments is denoted as *p < 0.05, **p <0.01, ***p < 0.001.

From metagenomic assembly, 300 high‐quality metagenome‐assembled genomes (MAGs) were reconstructed, with 237 carrying ARGs, VFGs, and mobile genetic elements (MGEs). ARG‐carrying MAGs spanned 16 genera, dominated by *Gordonia* (75.14%) and *Microbacterium* (8.34%) (Figure 2a,b). Exposure to increasing diversity of pharmaceuticals significantly enhanced the abundance of these composite carriers (*p* = 0.042, Kruskal–Wallis test, Figure 2b) in collembolan gut, with *Gordonia* exhibiting strong enrichment under six types of pharmaceutical conditions (*p* = 0.010, Kruskal–Wallis test, Figure 2c). Among *Gordonia* MAGs, which harbored 428 ARGs, the most prevalent resistance types were tetracycline (71.96%), rifamycin (21.03%), quinolone (3.04%), and multidrug resistance (2.34%) (Figure 2d). Furthermore, the number of CAZyme genes carried by *Gordonia* was significantly positively correlated with the number of ARGs (*p* = 0.002, Figure 2e), suggesting a link between metabolic versatility and resistance potential.

### 2.3 Potential Mechanisms Driving ARG Enrichment in Collembolan Guts

**FIGURE 3:**
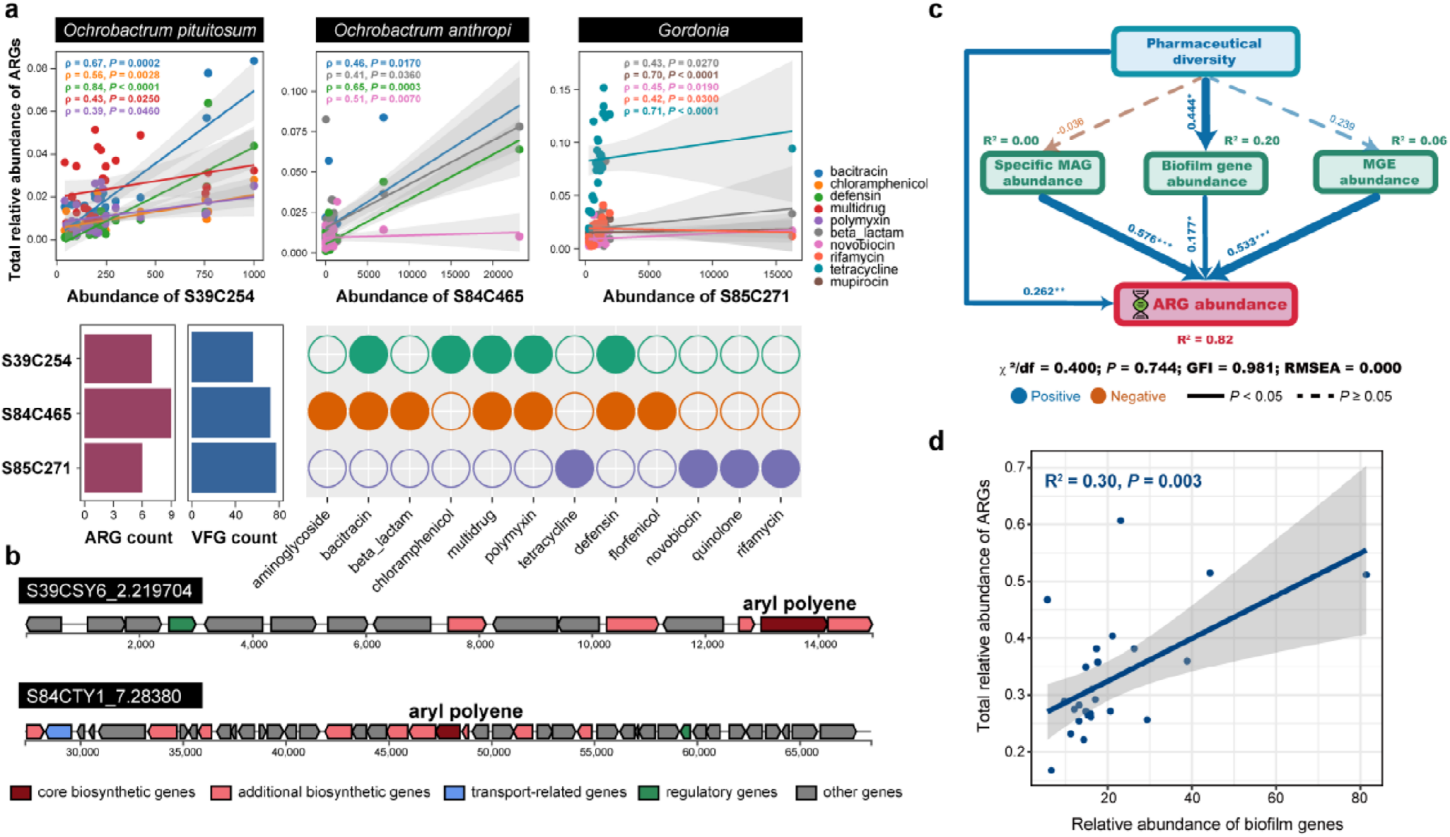
Biofilm formation is associated with enhanced ARG proliferation in collembolan gut. (a) Correlation between abundance of specific MAGs and total relative abundance of ARG types in gut samples (*n* = 27), and carried ARG and VFG information including gene counts and ARG types. Shaded bands represent 95% confidence intervals from linear regression. (b) Alignment of aryl polyene BGC from the genome of S39C254 and S84C465. The genes were colored according to the antiSMASH prediction. (c) Structural equation model (SEM) evaluating the direct and indirect effects of pharmaceutical diversity, abundances of key MAGs (S39C254, S84C456, S85C271), biofilm‐associated gene abundance, and MGE abundance on ARG abundance in all gut samples (*n* = 27). Blue and orange arrows indicate positive and negative effects, respectively. Solid and dotted lines show significant and insignificant effects, respectively. Path coefficients are shown next to the arrows, and R^2^ denotes the proportion of variance explained for each variable. Significance levels of regression weight are calculated using the two‐sided t‐test and denoted as *p < 0.05, **p < 0.01, ***p < 0.001. (d) Correlation between relative abundance of biofilm genes and ARGs in gut samples (*n* = 27). Shaded bands represent 95% confidence intervals from linear regression.

Three MAGs, *Ochrobactrum pituitosum* (S39C254), *Ochrobactrum anthropi* (S84C465), and *Gordonia* (S85C271), were significantly associated with the increasing abundance of multiple ARG types (*p* < 0.05, Figure 3a) in collembolan gut. These MAGs co‐carried diverse ARGs and VFGs, including resistance to polymyxin and tetracycline, and multidrug efflux pumps (Figure 3a). Biosynthetic gene cluster (BGC) analysis revealed a shared Aryl polyene cluster, commonly associated with biofilm production (Figure 3b). KEGG‐based functional profiling indicated that an increasing diversity of pharmaceuticals led to upregulated biofilm‐related pathways, including quorum sensing, ABC transporters, flagellar assembly, and biofilm formation (Figure S2a). Compared to the control, exposure to six types of pharmaceuticals significantly increased biofilm gene abundance in collembolan guts (*p* < 0.05, Wilcoxon test, Figure S2b).

To elucidate the direct and indirect effects of the diversity of pharmaceuticals, specific MAG abundance, biofilm gene abundance, and MGE abundance on total ARG abundance in the collembolan gut, we constructed a structural equation model (Figure 3c). The SEM explained 82% of the variance, revealing that diversity of pharmaceuticals (β = 0.262, *p* = 0.006), specific MAG abundance (β = 0.576, *p* < 0.001), biofilm gene abundance (β = 0.177, *p* = 0.050), and MGE abundance (β = 0.533, *p* < 0.001) directly and positively contributed to increased ARG abundance. Additionally, diversity of pharmaceuticals directly promoted biofilm gene enrichment (β = 0.444, *p* = 0.012). Linear regression further confirmed significant positive associations between biofilm gene (*p* = 0.003, R^2^ = 0.30, Figure 3d), MGE abundance (*p* = 0.006, R^2^ = 0.27, Figure S3), and ARG abundance.

### 2.4 Increasing Diversity of Pharmaceuticals Enhances Biofilm Formation and Proteomic Responses in Collembolan Gut Cultivable Bacteria

**FIGURE 4:**
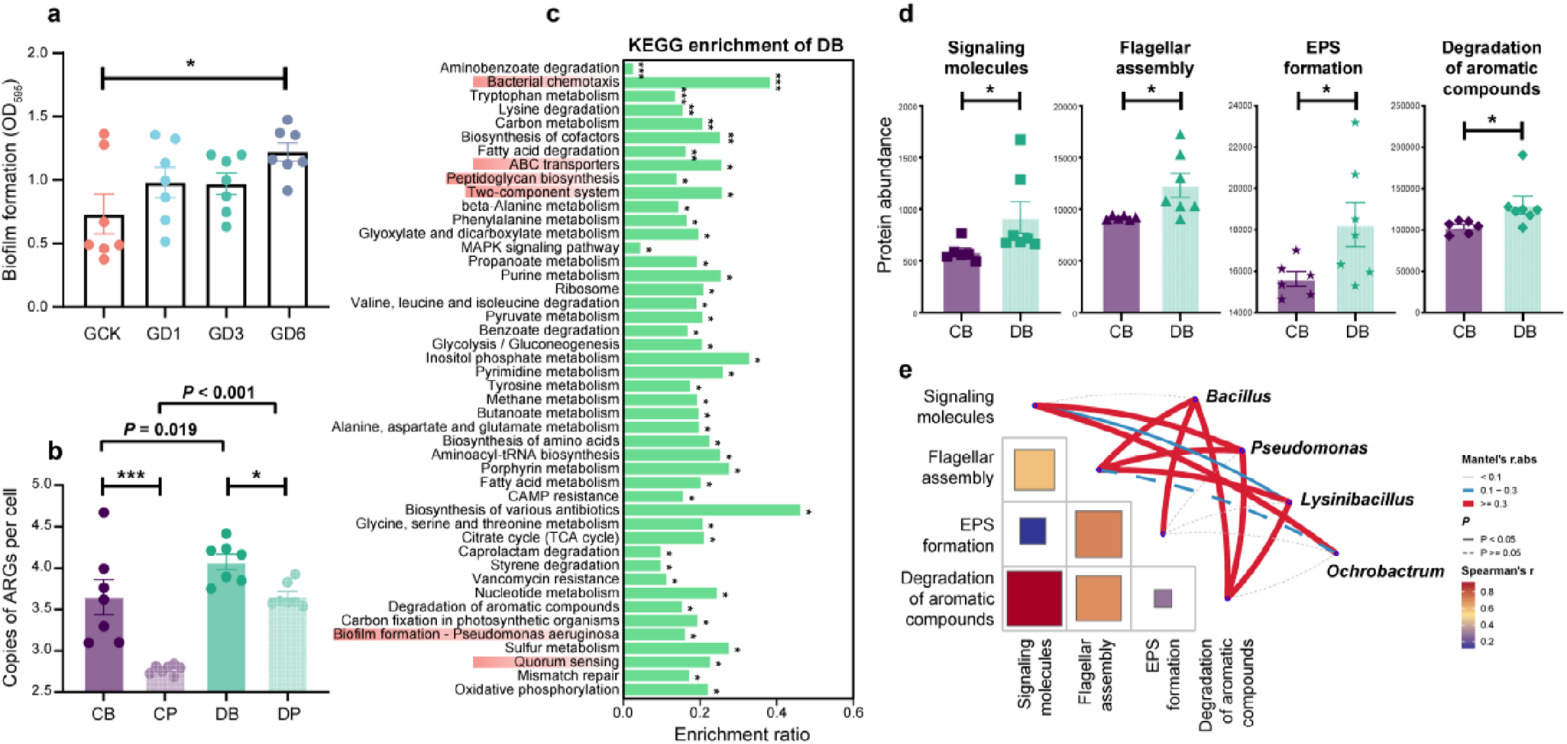
Increasing diversity of pharmaceuticals enhances biofilm formation and enriches ARGs within biofilm‐associated microbiome, validated by in vitro assays using collembolan gut isolates. (a) Biofilm biomass (OD595) of culturable collembolan gut microbiomes under increasing pharmaceutical diversity exposure (GCK: control; GD1, GD3, GD6: exposure to 1, 3, and 6 drugs, respectively; *n* = 7 per group). (b) Comparison of ARG abundance in biofilm versus planktonic bacterial communities under control and six‐pharmaceutical mixture exposure (CB: control biofilm; CP: control planktonic; DB: biofilm under pharmaceutical exposure; DP: planktonic under pharmaceutical exposure; *n* = 7 per group). (c) KEGG pathway enrichment analysis of biofilm communities exposed to the six‐pharmaceutical mixture (DB). KEGG pathways highlighted in red are associated with biofilm formation. (d) Differential abundance of key biofilm formation‐associated proteins between control and six‐pharmaceutical exposure treatments. (e) Correlations between dominant metabolically active bacterial genera and biofilm formation‐associated proteins within the biofilm matrix (*n* = 14). Solid lines represent significant correlations, while dashed lines indicate non‐significant correlations. Edge width and color corresponds to the Mantel correlation coefficient (*r*), and pairwise correlations are color‐coded based on Spearman's *r*. Error bars represent mean ± standard error (SE). Differences among treatments were evaluated using the Kruskal–Wallis test or thtwo‐sided Wilcoxon test. Significance among treatments is denoted as *p < 0.05, **p < 0.01, ***p < 0.001.

Exposure to a high diversity of pharmaceuticals significantly promoted biofilm formation in cultivable bacteria isolated from collembolan guts (GD6 vs. GCK: *p* = 0.026, Kruskal–Wallis test, Figure 4a). Biofilm formation positively correlated with increasing diversity of pharmaceuticals (*p* = 0.009, Figure S4a). Across treatments, ARG abundance was consistently higher in biofilm‐associated bacteria than in their planktonic counterparts under both control (*p* < 0.001, Kruskal–Wallis test) and high pharmaceutical diversity conditions (*p* = 0.023, Kruskal–Wallis test) (Figure 4b). Moreover, exposure to six pharmaceuticals significantly increased ARG abundance in both bacterial biofilms (*p* = 0.019, Kruskal–Wallis test) and planktonic populations (*p* < 0.001, Kruskal–Wallis test) compared to the control (Figure 4b). Notably, total ARG abundance in biofilms was strongly correlated with the extent of biofilm formation (*p* = 0.002, Figure S4b).

**FIGURE 5:**
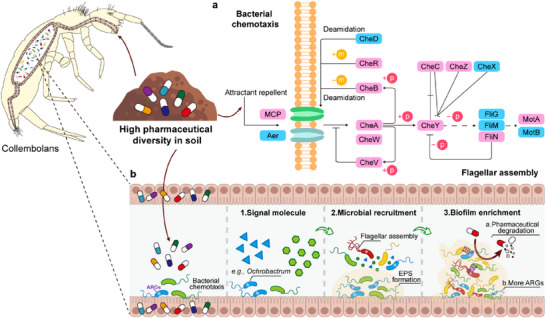
Mechanisms of pharmaceutical‐enhanced biofilm formation in collembolan gut. (a) KEGG pathway map of bacterial chemotaxis, with key proteins upregulated under six‐pharmaceutical mixture exposure highlighted in pink. (b) Conceptual model depicting the pharmaceutical‐enhanced biofilm formation process: (1) chemotaxis of gut microbes toward aromatic compounds; (2) initial colonization by *Ochrobactrum* and signaling molecule secretion; (3) recruitment of secondary colonizers with upregulation of flagellar assembly and extracellular polymeric substance (EPS) synthesis; and (4) biofilm maturation enriched with ARGs.

Proteomic analysis identified 15 293 proteins within the biofilm communities. Exposure to six types of pharmaceuticals induced substantial proteomic shifts, with 2787 proteins upregulated and 2678 downregulated relative to controls (fold change [FC] ≥ 1, *p* < 0.05, Figure S5). KEGG pathway enrichment revealed significant upregulation of key biofilm‐associated processes, including bacterial chemotaxis, ABC transporters, peptidoglycan biosynthesis, two‐component systems, quorum sensing, and biofilm formation (*p* < 0.05, Benjamini test, Figure 4c). Functionally, we classified biofilm‐related proteins into four categories: signaling molecules, flagella assembly, extracellular polymeric substance (EPS) formation, and degradation of aromatic compounds. All categories were significantly more abundant under exposure to six types of pharmaceuticals than in controls (*p* < 0.05, Wilcoxon test, Figure 4d). Taxonomic assignment linked signaling molecule production to metabolically active *Ochrobactrum*, while flagella assembly, EPS formation, and aromatic compound degradation were primarily associated with active *Bacillus*, *Pseudomonas*, and *Lysinibacillus*, taxa abundant under high pharmaceutical diversity (*p* < 0.05, Mantel test, Figure 4e, Figure S6). In addition, typical bacterial chemotactic proteins showed marked enhancement in response to pharmaceuticals, including (1) chemoeffector sensing via methyl‐accepting chemotaxis proteins (MCPs, *p* = 0.001, Wilcoxon test), (2) signal transduction through the CheA‐CheY two‐component system (*p* < 0.05, Wilcoxon test), and (3) downstream activation of flagellar motor proteins (FliN: *p* = 0.008; MotA: *p* = 0.001, Wilcoxon test) (Figure 5a).

### 2.5 Effects of Warming in the Collembolan Gut

Under warming, ARG abundance in the collembolan gut was inherently elevated (*p* = 0.006, Two‐sided Wilcoxon test, Figure S7a), leading to a paradoxical decline in ARG abundance upon pharmaceutical exposure relative to the non‐exposed control. For ARG subtypes, warming notably expanded the aminoglycosides, β‐lactam, chloramphenicol, MLS, multidrug, and polymyxin resistance (*p* < 0.05, Wilcoxon test, Figure S7b). The relationship between the diversity of pharmaceuticals and gene abundance was temperature‐dependent. At 20°C, increasing diversity of pharmaceuticals significantly elevated ARG (*p* = 0.012, Figure S8a) and VFG (*p* < 0.001, Figure S8b) abundance, while MGE abundance showed a non‐significant increasing trend (*p* = 0.325, Figure S8c). In contrast, at 23°C, a nonlinear (quadratic) response emerged, with peak ARG, VFG, and MGE abundances occurring at both the control and high‐diversity (six types of pharmaceuticals) treatments (*p* < 0.05, Figure S8b,d,e). After excluding controls to isolate pharmaceutical effects under warming, significant positive correlations were observed between the diversity of pharmaceuticals and ARG (*p* = 0.031, Figure S8d) and MGE (*p* = 0.041, Figure S8f) abundance. Although VFG abundance increased with the diversity of pharmaceuticals, this trend was not statistically significant (*p* = 0.245, Figure S8e).

Warming also reshaped collembolan gut microbial community structure. Warming significantly altered both ARG composition (*p* = 0.003, PERMANOVA test, Figure S9a) and microbial community structure (*p* = 0.003, PERMANOVA test, Figure S9b), with a strong correlation between the two (*p* < 0.001, Figure S9c). Several MAGs, notably those assigned to *Myroides* and *Cutibacterium humerusii*, were strongly associated with multiple ARGs (*p* < 0.05; Figure S9d) in collembolan gut, indicating key microbial contributors to ARG propagation under warming.

## 3 Discussion

The increasing diversity of pharmaceuticals represents a growing and underappreciated ecological risk, yet its impacts on non‐target soil organisms remain poorly understood. While previous research has established that individual pharmaceutical compounds drive resistance in bulk soils, and recent work has recognized the role of contaminant diversity in soil systems like microplastics [17], our results demonstrate that pharmaceutical diversity significantly alters the gut antibiotic resistome of collembolans. Notably, exposure to a greater number of pharmaceuticals at environmentally relevant concentrations substantially increased the abundance of ARGs in the collembolan gut, a pattern not observed in the surrounding soil. Due to their feeding on soil detritus, collembolans may ingest large quantities of humus‐bound pharmaceuticals [38], potentially contributing to these elevated ARG levels. These findings support our hypothesis that the gut microbiome of soil fauna is more sensitive to pharmaceutical diversity than bulk soil, acting as a significant early‐responder reservoir for ARGs [5, 39]. It should be noted as a limitation that the specific uptake rates and internal bioaccumulation of individual pharmaceuticals by the collembolan host were not analytically quantified in this study. Future toxicokinetic assessments are needed to precisely link the internal chemical burden of specific compounds with gut microbiome dynamics. Importantly, ARGs enriched in the collembolan gut can propagate across trophic levels and re‐enter the soil via various ecological interactions [6, 40]. Consequently, conventional soil ARG monitoring may lag behind these in vivo microbial changes; by the time resistance becomes evident in bulk soil, it may have already disseminated extensively through animal populations. These insights highlight the necessity of integrating both sensitive biological reservoirs (i.e., soil fauna) and chemical diversity into comprehensive antimicrobial resistance surveillance strategies.

In our study, a prominent shift in the collembolan gut resistome under high pharmaceutical diversity was the marked enrichment of nonspecific multidrug‐resistant classes mediated by efflux pumps, which increased over threefold. Among these, several Rank 1 risk ARGs including *mdtE*, *emrB*, and *tolC* were significantly elevated. Bacterial multidrug resistance efflux systems are pivotal in conferring resistance not only to antibiotics but also to a broad spectrum of xenobiotics, including heavy metals and metabolic byproducts [41, 42]. Supporting this broad‐spectrum defense, previous research has demonstrated the facilitatory effect of carbamazepine on microbial efflux systems in the collembolan gut [39], and certain bacteria are known to actively expel NSAIDs like ibuprofen via these pathways [43]. Mechanistically, genes such as *mdtE* and *emrB* (an RND family member) encode versatile pumps capable of exporting diverse compounds ranging from clinical antibiotics to environmental pesticides [44, 45]. Furthermore, *tolC* often co‐expresses with these pumps, forming a cooperative network that expels various substrates and contributes to cross‐resistance against multiple environmental stressors [46, 47]. While providing a general defense against pharmaceutical mixtures, this efflux‐mediated resistance simultaneously creates a genetic reservoir for cross‐resistance that could potentially compromise the efficacy of clinically important antibiotics. This underscores the cascading ecological consequences of pharmaceutical pollution: increasing chemical diversity not only elevates the overall ARG load but specifically enriches for the most problematic multidrug‐resistant variants.

In addition to the role of efflux pumps, the increasing diversity of pharmaceutical compounds creates heightened selective pressure on the collembolan gut microbial communities. Our study found that the abundance of bacteria carrying ARGs, VFGs, and MGEs increased significantly following exposure to multiple pharmaceuticals. This suggests that these bacteria are adapting to chemical selection pressures and may play critical roles in microbial resistance evolution within the collembolan gut niche [48, 49]. Among these bacteria, *Gordonia* was the most responsive genus, increasing in abundance and carrying multiple ARG types under high pharmaceutical diversity. *Gordonia* species are ecologically significant due to their ability to degrade a wide range of compounds, including substituted and unsubstituted hydrocarbons, toxic environmental pollutants, and xenobiotics [50]. Notably, some *Gordonia* species have been shown to degrade carbamazepine [51], highlighting their potential role in pharmaceutical degradation. A significant positive correlation between *Gordonia* CAZyme genes and ARGs suggests that metabolic flexibility may be linked to resistance proliferation. These traits underscore the dual role in pollutant degradation and resistance dissemination, making *Gordonia* a keystone taxon to consider in environments increasingly polluted with pharmaceuticals.

Importantly, our results demonstrated that the significant enrichment of biofilm formation driven by increasing pharmaceutical diversity was a key mechanism in elevating ARG abundance in the collembolan gut. While biofilms are well recognized as a general strategy for bacteria to resist individual stressors in laboratory cultures [52, 53], our findings provide evidence that chemical complexity governs biofilm‐mediated resistance in animal microbiomes. Biofilms, which are formed by a complex and diverse community of microorganisms embedded in an extracellular matrix composed of polysaccharides, exudates, and detritus [54], provide a protective environment that enhances bacterial survival and facilitates the exchange of genetic material (including ARGs), thereby promoting the persistence and spread of resistance [55]. In soil, biofilm‐associated effects are often masked by the complexity of microbial assemblages. In contrast, the collembolan gut offers a clearer signal, as most microorganisms exist in biofilm form. Aryl polyenes are a group of natural products widespread in bacteria [56]. Their BGCs were enriched in the bacterial MAGs that significantly positively correlated with multiple ARGs in our study. These compounds can protect bacteria from reactive oxygen species and promote biofilm formation [57, 58]. Previous studies have shown that heterologous expression of aryl polyene BGCs in *Escherichia coli* strongly promotes biofilm formation [57]. Similarly, bacteria carrying aryl polyene BGCs have been observed to consistently form a continuous biofilm on the inner wall of the honeybee ileum [59]. Thus, microbes adapted to increasing pharmaceutical diversity may have enhanced ARG abundance in the collembolan gut by promoting biofilm formation, consistent with our hypothesis. Building upon these findings, we isolated culturable microbes from the collembolan gut and established in vitro biofilm culture systems to investigate the pharmaceutical diversity‐biofilm relationship. Our results demonstrated that elevated pharmaceutical diversity significantly enhanced biofilm formation, with a concomitant increase in ARG abundance compared to control conditions. Notably, biofilm bacteria maintained significantly higher ARG abundance than their planktonic counterparts, suggesting that the biofilm matrix provides an optimal microenvironment for ARG maintenance and propagation in the collembolan gut, adapting to increasing pharmaceutical diversity.

To elucidate the mechanisms underpinning ARG enrichment under a high diversity of pharmaceuticals, we performed metaproteomic sequencing of biofilm‐associated microbial communities. Our results revealed a significant upregulation of chemotaxis and signaling pathways related to biofilm development. In contrast to single‐drug exposure, multiple pharmaceuticals at environmental concentrations may interact synergistically [60, 61], mimicking the amplified chemical gradients typically observed in high‐concentration single‐contaminant exposure. In our study, this phenomenon likely triggered enhanced bacterial chemotaxis through the activation of methyl‐accepting chemotaxis proteins (MCPs). MCPs are the most common receptors in bacteria and archaea [62]. These transmembrane proteins enable precise sensing of chemical stimuli and amplify signaling, facilitating adaptive responses to environmental changes [63]. Bacterial chemotaxis toward aromatic compounds has been well‐documented, with many microbes exhibiting directed movement along aromatic compound gradients [64, 65]. Notably, many of the pharmaceuticals in this study contain aromatic structures, which may explain the observed upregulation of MCPs under an increasing diversity of pharmaceuticals. Following signal amplification through the chemotaxis cascade, the CheA‐CheY regulatory system detects and processes the enhanced signaling input [66]. Our proteomic analysis revealed that exposure to a high diversity of pharmaceuticals significantly enriched multiple Che proteins, with CheR and CheV showing a 2.4‐fold increase and CheA and CheY demonstrating a 1.0‐fold upregulation compared to control conditions. This substantial enrichment of the chemotaxis machinery components suggests a robust adaptive response to complex pharmaceutical mixtures. The CheA‐CheY regulatory system is instrumental in biofilm development, mediating flagella‐driven motility and directing bacteria toward favorable microenvironments for biofilm establishment [67, 68]. Deletion of *cheA*‐*R* or *fliM* in *Azorhizobium caulinodans* has been shown to significantly reduce biofilm biomass [69], underscoring the regulatory role of the Che signaling pathway in biofilm formation. Furthermore, a high diversity of pharmaceuticals significantly enriched proteins involved in signaling, EPS formation, and aromatic compound degradation. This suggests a coordinated microbial response, where chemotaxis‐driven bacterial recruitment facilitates biofilm formation (Figure 5b). *Ochrobactrum* may mediate early‐stage biofilm development by secreting signaling molecules, promoting the aggregation of EPS‐producing taxa such as *Bacillus* and *Pseudomonas*. The mature biofilm, enriched in ARGs, likely enhances microbial resilience by degrading aromatic compounds, mitigating environmental stress. *Bacillus* spp. are aerobic or facultatively anaerobic bacteria capable of synthesizing and secreting diverse EPS with multifunctional roles, including insecticidal, antimicrobial, nitrogen‐fixing, and probiotic activities [70]. Cellulose‐based EPS have been extensively reported in *Pseudomonas* species [71], highlighting their structural and functional significance in microbial adaptation and biofilm formation. These findings reveal a complex microbial adaptation strategy, where chemotaxis‐driven biofilm formation under a high diversity of pharmaceuticals enhances ARG retention and environmental resilience in the collembolan gut, highlighting the critical role of biofilm dynamics in antimicrobial resistance dissemination.

Moreover, global change factors such as warming can significantly alter the response of the collembolan gut resistome to an increasing diversity of pharmaceuticals. While conventional models utilizing constant warming have established temperature as a driver of ARG dissemination, our simulated diurnal temperature fluctuations reveal a more complex ecological response. Our study found that warming alone substantially promoted the abundance of gut ARGs in collembolans unexposed to pharmaceuticals, driven primarily by the genus *Myroides*. *Myroides* species are commonly found in various environments and are known for their resistance to a wide range of antibiotics [72]. However, under warming conditions, the promotion of gut ARGs driven by increasing pharmaceutical diversity was masked. This outcome may stem from two concurrent processes: first, warming alone strongly enriched gut ARGs; second, the combined stress of warming and multi‐pharmaceutical exposure may have exceeded the adaptive capacity of the gut microbiome, leading to microbial community disruption and reduced resistance gene transmission. For instance, co‐exposure to multiple antibiotics significantly reduced the total relative abundance of ARGs in plant roots and led to a marked decrease in *intI1* levels compared to single sulfamethoxazole exposure [73]. This issue warrants attention because global warming and the increasing release of pharmaceuticals into the environment are co‐occurring phenomena. This masking effect complicates the accurate assessment of ecological risks associated with chemical mixtures and hinders the establishment of effective regulatory standards under future climate scenarios.

Collectively, our study identifies pharmaceutical diversity as an independent driver of ARG enrichment in the collembolan gut, primarily through the expansion of nonspecific efflux pumps, the increase in *Gordonia* abundance, and biofilm formation. By demonstrating the synergistic effects of chemical diversity and warming, this work highlights the need for a more comprehensive assessment of resistance risks in soil animal systems. Our findings suggest that monitoring pharmaceutical diversity, in addition to concentration, is essential for understanding the ecological impacts of pharmaceutical pollution under global change. Future research should aim to delineate the in vivo molecular interactions within these complex host microbiomes and evaluate the potential for trophic transfer of these enriched ARG reservoirs to higher predators. Moreover, translating these microcosm‐based findings to field‐scale investigations will be crucial for validating the broader ecological consequences of chemical complexity.

## 4 Materials and Methods

### 4.1 Test Animals, Pharmaceuticals, and Experimental Soil Preparation

To investigate the impact of an increasing diversity of pharmaceuticals on the gut microbiome and resistome of soil animals, we used the model soil‐dwelling collembolan *Folsomia candida*. This species was originally obtained from the University of Aarhus, Denmark, and has been continuously cultured in our laboratory for over a decade in accordance with the guidelines of the Organization for Economic Co‐operation and Development (OECD). Collembolans were maintained on a 0.5 cm thick substrate composed of a 9:1 (w/w) mixture of moist plaster of Paris (USG Boral, China) and activated carbon. The culture dishes were supplemented twice weekly with yeast (Angel Yeast Co., Ltd, China) as a food source, and distilled water was added weekly to maintain substrate moisture. To obtain age‐synchronized individuals for the experiment, 50–60 adult *F. candida* were transferred to fresh substrates for egg laying. After sufficient egg deposition, adults were removed. Juveniles were collected three days post‐hatching and cultured on fresh substrate until they reached 10–12 days of age. These synchronized juveniles were then exposed to pharmaceutical‐spiked soil for experimental treatments.

Seven commonly used non‐antibiotic human pharmaceuticals were selected for this study: carbamazepine (C_15_H_12_N_2_O, CAS No.: 298‐46‐4, purity: 99.5%), thioridazine (C_21_H_26_N_2_S_2_, CAS No.: 50‐52‐2, purity: 98.5%), ibuprofen (C_13_H_18_O_2_, CAS No.: 15687‐27‐1, purity: 99%), gemfibrozil (C_15_H_22_O_3_, CAS No.: 25812‐30‐0, purity: 99.5%), cetirizine (C_21_H_25_ClN_2_O_3_, CAS No.: 83881‐51‐0, purity: 99%), metformin (C_4_H_11_N_5_, CAS No.: 657‐24‐9, purity: 99%), and citalopram (C_20_H_21_FN_2_O·HBr, CAS No.: 5972‐33‐8, purity: 98%). These specific compounds were chosen based on their widespread global usage, high detection frequencies, and frequent co‐occurrence as complex mixtures in agricultural soils and wastewater effluents [11, 74, 75]. The selection of non‐antibiotic pharmaceuticals was designed to test whether the chemical diversity of non‐target pollutants—rather than the direct selection pressure of traditional antibiotics—independently drives ARG enrichment. Furthermore, a substitutive gradient design was employed to strictly isolate the effect of compound diversity from total chemical concentration. The final total pharmaceutical concentration in soil was 1.2 mg/kg, corresponding to the upper range of reported environmental levels [74]. In the highest‐diversity treatment (six pharmaceutical types), the average concentration of each individual compound was set at 0.2 mg/kg [75], consistent with environmentally relevant concentrations. This ensures that the total chemical burden remained comparable across all treatments, allowing the ecological effects of compound diversity to be unambiguously disentangled from those of dose‐dependent responses.

Soil was collected from the topsoil layer (0–20 cm) near Beilun Township, Zhejiang Province, China (pH 6.8, electrical conductivity: 90 µS/cm, total organic carbon: 0.75%). The collected soil was air‐dried in the shade, and visible gravel, plant residues, and other debris were removed. The cleaned soil was then homogenized and sieved through a 2 mm mesh for experimental use. Stock solutions of the selected pharmaceuticals were prepared by dissolving each compound in a small volume of 100% ethanol, followed by dilution with ultrapure water. These solutions were thoroughly mixed with dry soil to achieve a final total pharmaceutical concentration of 1.2 mg/kg dry soil. The spiked soil was placed in a ventilated chamber for 24 h to allow complete evaporation of ethanol. Afterward, the soil was freeze‐dried, ground, and sieved again through a 2 mm mesh. This sieving and homogenization step is a standard requirement for soil ecotoxicity tests (OECD 232 guideline) to guarantee the homogeneous distribution of the spiked pharmaceuticals. It effectively eliminates localized concentration hotspots, thereby strictly controlling the chemical exposure variable. Soil moisture was then adjusted to 40% of the water‐holding capacity using ultrapure water. Before use, all treated soils were pre‐incubated under climate‐controlled conditions for one week. Control soils were prepared following the same procedures, with ultrapure water added in place of the pharmaceutical stock solutions.

### 4.2 Experimental Design

Microcosm systems were established using 50 mL glass beakers (4 cm inner diameter × 6 cm height), each filled with 40 g (wet weight) of treated soil and 20 age‐synchronized *F. candida* individuals. Pharmaceuticals were added to soil at a total concentration of 1.2 mg/kg dry weight. Four levels of pharmaceutical diversity were tested: 0 (control), 1, 3, and 6 different pharmaceutical types. Because our experimental pool consisted of 7 distinct pharmaceuticals, each treatment level was mathematically required to be replicated seven times (*n* = 7, Figure S10) to establish a fully balanced compositional matrix. For the single‐pharmaceutical treatment (D1), each of the seven drugs was tested individually (i.e., one single, different pharmaceutical added for each of the seven replicates of this level). To ensure that the observed ecological responses were driven by chemical diversity rather than the idiosyncratic toxicity of any specific compound (identity effect), we employed a fully balanced combinatorial design. For the three‐pharmaceutical treatment (D3), seven unique combinations were prepared using a balanced matrix, ensuring that each of the seven drugs was represented exactly three times across the seven replicates. For the six‐pharmaceutical treatment (D6), seven unique combinations were created using a “leave‐one‐out” approach, where each drug appeared exactly six times across the replicates. We did not include a seven‐pharmaceutical treatment, since there would be no compositional variability among the replicates of this treatment, as there was a pool of seven pharmaceuticals overall. Our design emphasizes the number of items, in this case, pharmaceuticals, added, rather than their identity or composition. Across all diversity levels, the total pharmaceutical concentration was held constant at 1.2 mg/kg dry soil, corresponding to 1.2 mg/kg for one pharmaceutical, 0.4 mg/kg for each of the three pharmaceuticals, and 0.2 mg/kg for each of the six pharmaceuticals. Microcosms were maintained under standard rearing conditions for *F. candida* (20°C, 75% relative humidity). To simulate climate warming, an additional fluctuating regime with a 3°C increase within a 24‐h cycle was applied using a climate chamber (Memmert ICP110, Germany) programmed with AtmoCONTROL software. The temperature gradually increased from 20°C to 23°C over 12 h and subsequently decreased back to 20°C during the following 12 h. This cycle was repeated continuously for 28 days, mimicking natural diurnal temperature variation rather than imposing constant thermal stress. In total, the experiment included 4 pharmaceutical diversity levels × 2 temperature regimes × 7 replicates, resulting in 56 microcosms. Soil moisture was maintained by biweekly addition of ultrapure water. After 28 days, collembolans were extracted via flotation, and representative soil samples were collected for DNA analysis.

### 4.3 DNA Extraction From Soil and Collembolan Samples

Collembolans were surface‐sterilized with 2% sodium hypochlorite for 10 s and rinsed five times with sterile phosphate‐buffered saline (PBS) to eliminate external bacterial contamination. Guts were dissected under sterile conditions using sterile forceps and transferred to 1.5 mL centrifuge tubes containing 20 µL proteinase K and 180 µL lysis buffer. Samples were homogenized in microcentrifuge tubes, and DNA was extracted using the DNeasy Blood and Tissue Kit (QIAGEN, Germany) following the manufacturer's instructions. For soil samples, total genomic DNA was extracted from a standardized 0.5 g aliquot—the optimal starting weight recommended to prevent column overloading and maximize cell lysis efficiency—using the FastDNA Spin Kit for Soil (MP Bio, USA) according to the manufacturer's instructions. DNA quantity and purity were assessed via NanoDrop ND‐1000 spectrophotometry (Thermo Fisher Scientific, USA) and 1% agarose gel electrophoresis. DNA samples were stored at −20°C until sequencing.

### 4.4 Isolation of Gut Bacteria and Biofilm Formation Experiment

To investigate biofilm formation under pharmaceutical exposure, gut bacteria were isolated from *F. candida* and subjected to controlled in vitro treatments. Forty adult collembolans (23–25 days old) were surface‐sterilized and dissected under aseptic conditions. Fresh gut tissues were suspended in 200 µL of sterile phosphate‐buffered saline (PBS) and homogenized using a sterile mortar and pestle. The homogenate was inoculated into 10 mL of Luria‐Bertani (LB) broth and incubated at 37°C with shaking at 180 rpm until reaching the logarithmic growth phase. The resulting bacterial culture was adjusted to an optical density of OD_595_ =  1.0.

For biofilm induction, 1 mL of bacterial suspension was transferred into 24‐well sterile polystyrene culture plates. Pharmaceutical treatments were applied to each well following the same combinations and total concentrations as described in the soil exposure experiment (final concentration: 1.2 mg/L). Cultures were incubated at 37°C with 100 rpm for 4 days to promote biofilm development both on the surfaces completely submerged in the liquid medium (solid–liquid interface) and at the liquid margin along the well edges (gas–liquid interface). This 4‐day incubation period was selected because the metabolic activity, structural features, and bacterial composition of the biofilm typically reach maturity and remain stable from 3 to 6 days in vitro [76]. Two sets of plates were prepared. In one set, biofilm biomass was quantified using crystal violet staining to assess overall biofilm formation. In the second set, mature biofilms were harvested from the control and high‐diversity pharmaceutical (D6) treatments using sterile cotton swabs. Collected biofilm samples were subjected to metagenomic and metaproteomic sequencing for downstream analysis of ARG profiles, community composition, and protein expression associated with pharmaceutical diversity.

### 4.5 Metagenomic Sequencing and Bioinformatics Analysis

DNA libraries were constructed with the NEBNext Ultra DNA Library Prep Kit (NEB, USA) and sequenced on the Illumina HiSeq4000 platform (PE150) by Majorbio Bio‐Pharm Technology (Shanghai, China). Approximately 10 Gb of raw reads were obtained per soil sample, and 50 Gb per gut sample. Low‐quality reads were removed using standard filtering criteria. For gut samples, host sequences were removed using Bowtie2 against four *F. candida* reference genomes (GCF_002217175.1, GCA_020920555.1, GCA_020923455.1, GCA_034694505.1) with “–very‐sensitive” mode.

High‐quality reads were assembled into contigs using MEGAHIT [77]. ORFs were predicted using Prodigal in metagenome mode on contigs ≥500 bp [78]. Redundant sequences were clustered with CD‐HIT (95% identity, 90% coverage) [79]. Reads were mapped back to the gene catalog with Bowtie2 [80], and gene abundance was quantified using TPM values via SAMtools [81]. Taxonomic assignment was performed using DIAMOND BLASTp (*e*‐value ≤ 1e^−5^) against the NCBI NR database, and taxonomic resolution was refined using MEGAN's lowest common ancestor algorithm [82].

Functional annotation was carried out using DIAMOND searches against KEGG [83], selecting hits with *e*‐values ≤ 1e^−5^ and HSP > 60 bits. The abundance of functional categories was derived from the sum of annotated gene abundances. ARGs were quantified via the ARGs‐OAP 3.2 pipeline based on the SARG database [84] and normalized to gene copies per cell. MGEs and VFGs were annotated using MobileOG [85] and VFDB [86], respectively, with a consistent filtering threshold (*e*‐value ≤ 1e^−7^, identity ≥ 80%, and alignment length ≥ 75%) applied across all gene types. High‐risk ARGs were classified according to a previously established reference list [87].

### 4.6 Metagenomic Binning and Functional Annotation

Metagenome‐assembled genomes (MAGs) were reconstructed using the MetaWRAP pipeline [88]. MAG quality was assessed using CheckM [89], retaining bins with completeness >50% and contamination <10%. Taxonomic classification and phylogenetic tree construction were performed with GTDB‐Tk [90], and results were visualized using Chiplot (https://www.chiplot.online/) [91]. ORFs were predicted using Prodigal and annotated via DIAMOND BLASTx searches against the SARG database. Alignments were performed in sensitive mode with thresholds of *e*‐value ≤ 1e^−7^ and identity ≥ 80%. MGEs and VFGs were identified by aligning MAG‐derived protein sequences against the MobileOG database using the same search parameters. Biosynthetic gene clusters (BGCs) were predicted using antiSMASH v6.0 with default settings [92], enabling the detection of both known and putative secondary metabolite clusters.

### 4.7 Metaproteomic Sequencing and Bioinformatics Analysis

Frozen samples were thawed on ice and suspended in BPP buffer. Cells were disrupted using a high‐flux tissue grinder (3 × 40 s cycles), followed by centrifugation (12 000 g, 4°C, 20 min). The supernatant was extracted with an equal volume of Tris‐saturated phenol, vortexed for 10 min at 4°C, and centrifuged under the same conditions. The phenol phase was re‐extracted with BPP buffer, vortexed, and centrifuged again. The final phenol phase was mixed with 5 volumes of pre‐chilled ammonium acetate in methanol and incubated overnight at −20°C. Protein precipitates were washed twice with 90% cold acetone, then dissolved in lysis buffer (8 m urea, 1% SDS, protease inhibitor cocktail), sonicated on ice (2 min), and clarified by centrifugation. Protein concentration was quantified using the BCA Protein Assay Kit (Thermo Scientific), and integrity was verified by SDS‐PAGE. For digestion, 100 µg of protein was reduced with 10 mM TCEP (37°C, 1 h), alkylated with 40 mm iodoacetamide (room temperature, 40 min, dark), and centrifuged. The pellet was resuspended in 100 mM TEAB buffer and digested overnight at 37°C with trypsin (1:50 w/w). Peptides were vacuum‐dried, reconstituted in 0.1% TFA, desalted using HLB cartridges, and dried again. Peptide concentrations were measured with NanoDrop ND‐1000 spectrophotometry. Peptide identification was performed by aligning sequences against the custom metagenomic protein database using DIAMOND or BLASTp. High‐confidence matches were selected for protein identification. For quantification, DIA raw data were analyzed using Spectronaut v18 against the matched metagenomic database. Parameters included: Protein FDR ≤ 1%, peptide FDR ≤ 1%, peptide confidence ≥ 99%, and XIC width ≤ 75 ppm. Shared and modified peptides were excluded. Quantification was based on the summed peak areas of unique peptides, with at least one unique peptide required for protein identification.

Differentially expressed proteins (DEPs) were defined as those with a fold change >1.5 or <0.67 and *p* < 0.05 (two‐tailed *t*‐test). DEPs were annotated for functional classification using the KEGG database. KEGG pathway enrichment analysis of upregulated proteins in the high‐diversity group was conducted using the hypergeometric distribution with Fisher's exact test. False discovery rate (FDR) was controlled using the Benjamini–Hochberg (BH) procedure. KEGG pathways with FDR‐adjusted *p* values < 0.05 were considered significantly enriched.

### 4.8 Statistical Analysis

Statistical analyses were performed using SPSS v26.0 (IBM, Chicago, USA) and R v4.2.1. Data are presented as means or mean ± standard error (SE). The sample size was *n* = 7 biologically independent replicates per treatment level for soil samples, and *n* = 7 for collembolan gut samples (except for the GD1 gut group, where *n* = 6 due to amplification failure). Differences among pharmaceutical diversity treatments were assessed using the Kruskal–Wallis test and two‐sided Wilcoxon test. Principal Coordinates Analysis (PCoA) based on Bray–Curtis dissimilarity was applied to visualize differences in ARG composition using the “vegan” R package (v2.6‐4). Permutational multivariate analysis of variance (PERMANOVA) was used to quantify the proportion of variance explained by treatments. Linear regression analyses were conducted using the “ggplot2” package. Mantel tests with 999 permutations were used to assess correlations between the abundance matrices of metabolically active *Bacillus*, *Pseudomonas*, *Lysinibacillus*, and *Ochrobactrum* and biofilm‐associated proteins using the “linkET” package. Procrustes analysis was performed to examine the congruence between ARG profiles and bacterial community composition using the “vegan” package. To disentangle direct and indirect effects of pharmaceutical diversity on gut ARG abundance, we constructed a structural equation model (SEM) using AMOS v21.0 (IBM, Chicago, USA). The model incorporated pharmaceutical diversity, abundances of key MAGs (S39C254, S84C456, S85C271), biofilm‐associated gene abundance, and MGE abundance as predictors. Model fit was evaluated using the χ^2^ test, goodness‐of‐fit index (GFI), and root mean square error of approximation (RMSEA). Statistical significance was set at *p* < 0.05.

## Author Contributions

All authors contributed intellectual input and assistance to this study. D.Z. and Y.‐F.W. conceived and designed the experiments. Y.‐F.W., Y.‐N.W., and D.L. carried out the experiments. Y.‐F.W., J.‐Y.X. and F.‐Y.Q. analyzed the data. Y.‐F.W. and H.‐L.C. prepared the figures. Y.‐F.W. wrote the first draft, and H.‐J.L., E.T., M.‐C.R., Y.‐G.Z., M.Q., and D.Z. reviewed and commented on the paper. All authors read and approved the manuscript.

## Conflicts of Interest

The authors declare no conflicts of interest.

## Supporting information




**Supporting File**: advs75272‐sup‐0001‐SuppMat.docx.

## Data Availability

The sequencing data generated in this study have been deposited in the National Center for Biotechnology Information (NCBI) SRA database (https://www.ncbi.nlm.nih.gov/sra) under accession code PRJNA1265928.
