# Supplementary material for: Diversity of Pharmaceuticals Enhances Antibiotic Resistance in the Invertebrate Gut via Biofilm‐Mediated Mechanisms
Source: Adv Sci (Weinh). 2026 Apr 13:e18849. Online ahead of print. doi: 10.1002/advs.202518849 (PMC13334655; doi:10.1002/advs.202518849)
Supplement: Supplementary file 1 — Supporting File: advs75272‐sup‐0001‐SuppMat.docx. [file ADVS-9999-e18849-s001.docx]

Supplementary Materials for

**Diversity of Pharmaceuticals Enhances Antibiotic Resistance in the Invertebrate Gut via Biofilm-Mediated Mechanisms**

Yi-Fei Wang^1,2^, Ya-Ning Wang^1,3^, Da Lin^1,3^, Jia-Yang Xu^1,3^, Feng-Yuan Qi^3,4^, Hui-Ling Cui^4^, Hui-Jie Lu^5^, Min Qiao^4^, Edward Topp^6^, Dong Zhu^1,2*^, Matthias C. Rillig^7,8^, Yong-Guan Zhu^1,2,3,4^

^1^ State Key Laboratory of Regional and Urban Ecology, Ningbo Observation and Research Station, Institute of Urban Environment, Chinese Academy of Sciences, Xiamen 361021, China.

^2^ Zhejiang Key Laboratory of Pollution Control for Port-Petrochemical Industry, CAS Haixi Industrial Technology Innovation Center in Beilun, Ningbo 315830, China.

^3^ University of Chinese Academy of Sciences, Beijing 100049, China.

^4^ State Key Laboratory of Regional and Urban Ecology, Research Center for Eco-Environmental Sciences, Chinese Academy of Sciences, Beijing 100085, China.

^5^ Key Laboratory of Environment Remediation and Ecological Health, Ministry of Education, College of Environmental Resource Sciences, Zhejiang University, Hangzhou, Zhejiang 310058, China.

^6^ London Research and Development Centre (LRDC), Agriculture and Agri-Food Canada, London, Ontario N5V 4T3, Canada

^7^ Institute of Biology, Freie Universität Berlin, Berlin 14195, Germany.

^8^ Berlin-Brandenburg Institute of Advanced Biodiversity Research (BBIB), Berlin 14195, Germany.

* Corresponding Author: Dong Zhu

Email address: dzhu@iue.ac.cn

**This PDF file includes:**

Figs. S1 to S10


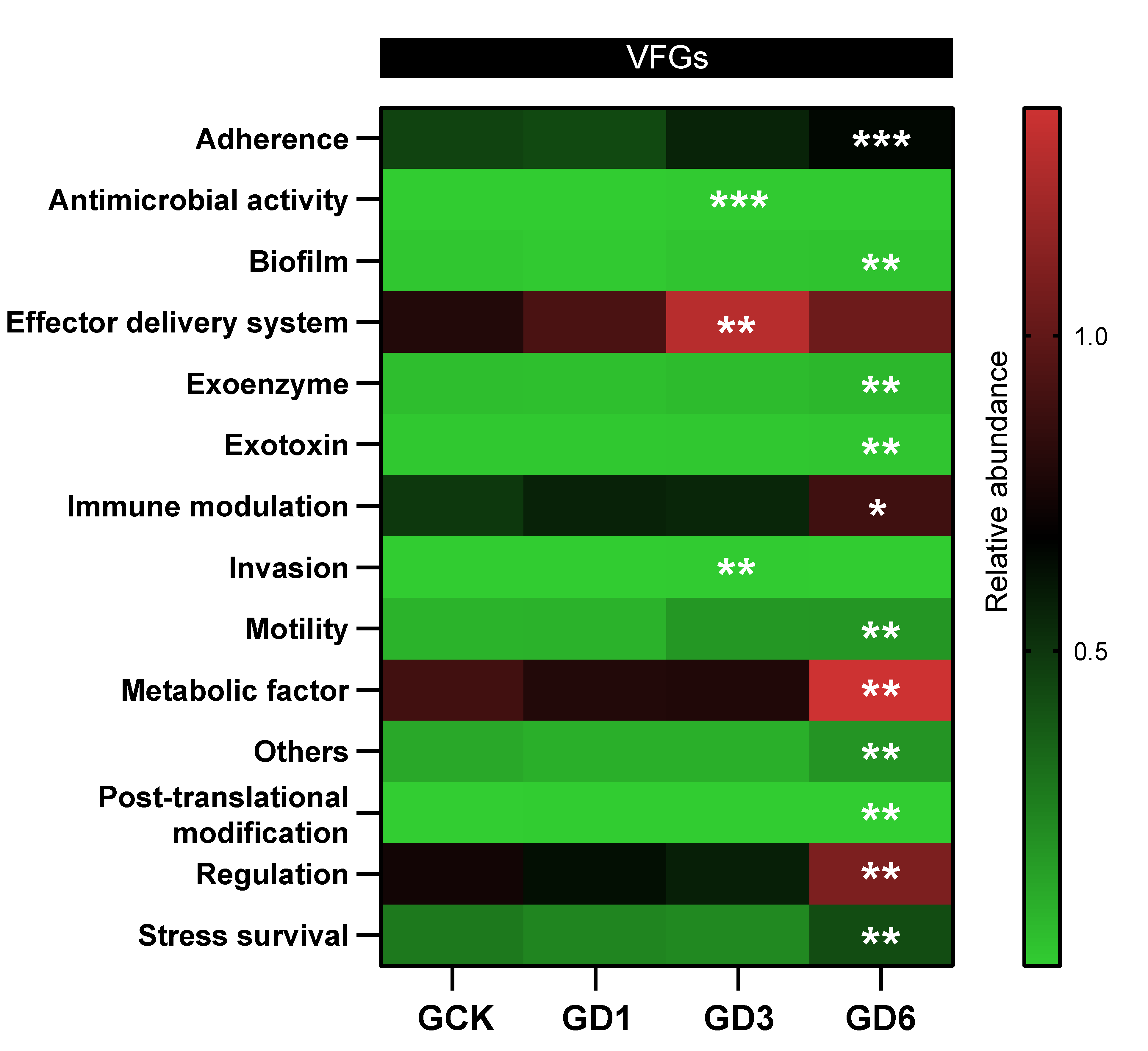


Fig. S1. Effects of increasing pharmaceutical diversity on VFG types in the collembolan gut.

Heatmap showing the relative abundance of different VFG types across collembolan gut samples (GCK: control; GD1, GD3, GD6: 1, 3, and 6 drugs, respectively; *n* = 7 per group, except GD1 where *n* = 6 due to one sample failing amplification).

Significance among treatments is denoted as * *P* < 0.05, ** *P* < 0.01, *** *P* < 0.001. The unit of genes is copies per cell.


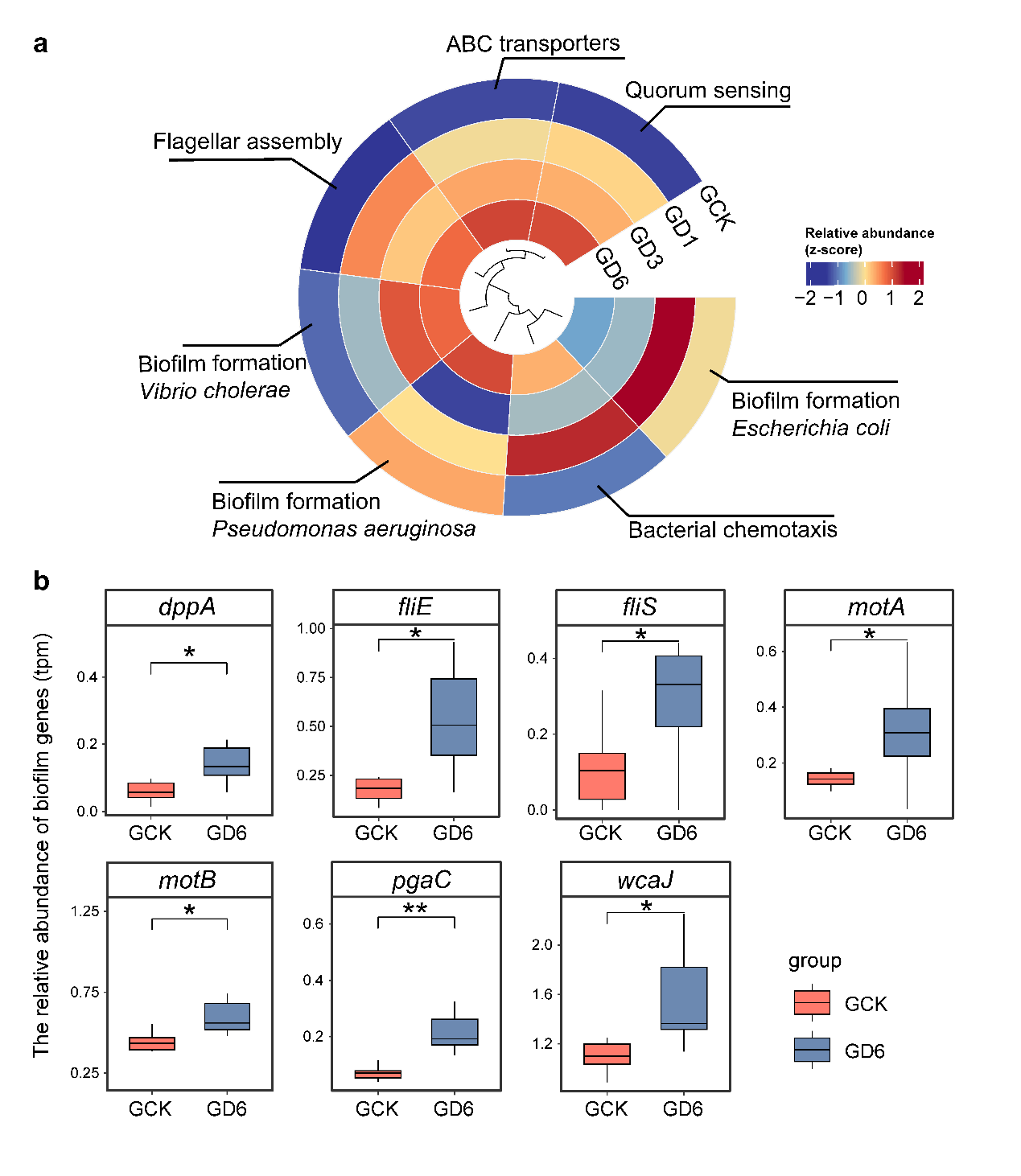


Fig. S2. Increasing diversity of pharmaceutical promotes enrichment of biofilm-related functions in collembolan gut microbiome.

a, Circular heatmap showing the relative abundance (z-score) of biofilm-related KEGG pathways across collembolan gut samples (*n* = 7 per group, except GD1 where *n* = 6 due to amplification failure).

b, Boxplots showing the relative abundance (TPM) of representative biofilm-associated genes across control (GCK) and high-diversity pharmaceutical exposure (GD6) groups.

Significance among treatments is denoted as * *P* < 0.05, ** *P* < 0.01, *** *P* < 0.001.


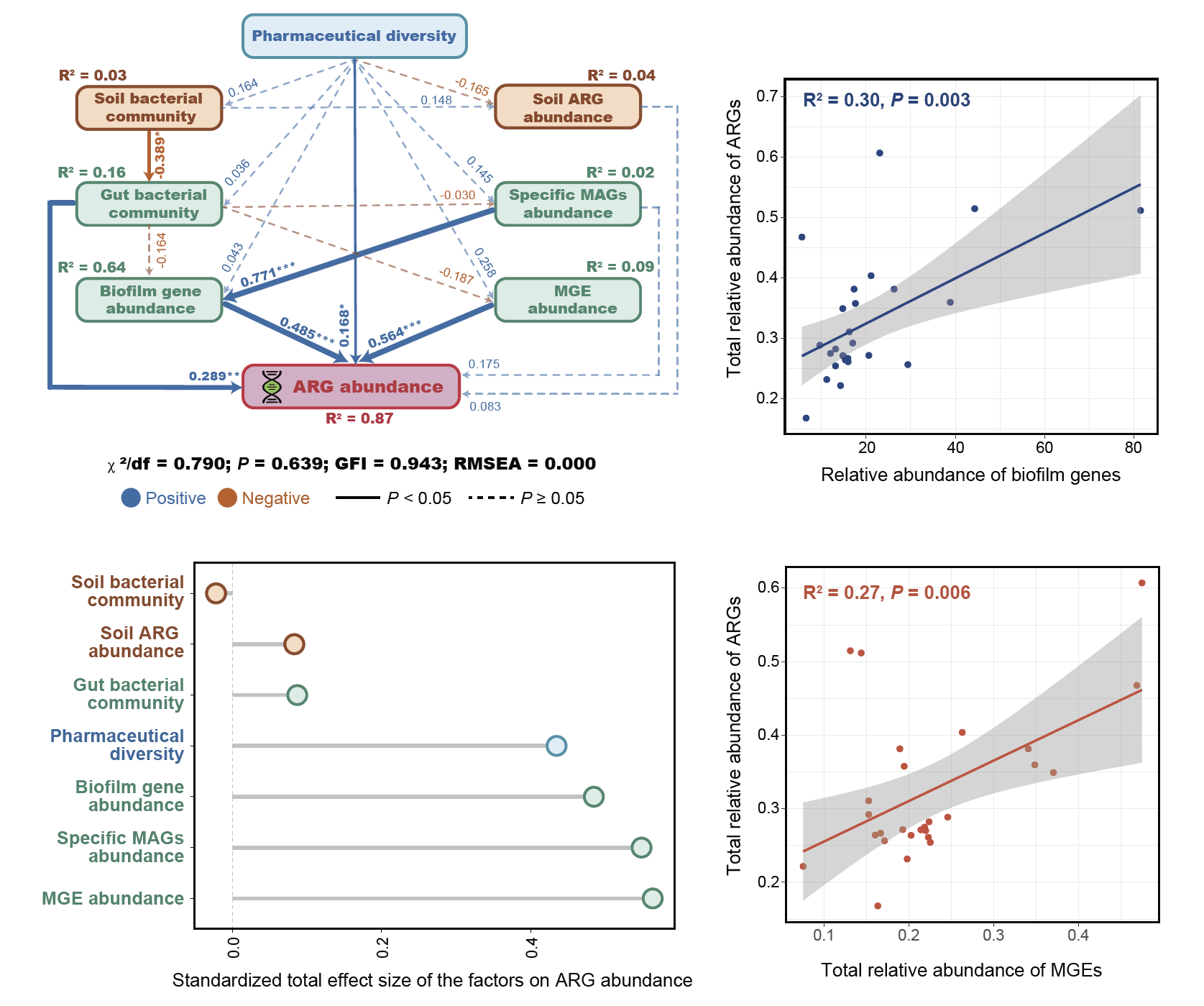


Fig. S3. Correlation between total relative abundance of MGEs and ARGs in collembolan gut samples.

The scatter plot shows the relationship between total MGE and ARG abundance across gut samples (*n* = 27). Shaded bands indicate 95% confidence intervals derived from linear regression analysis.


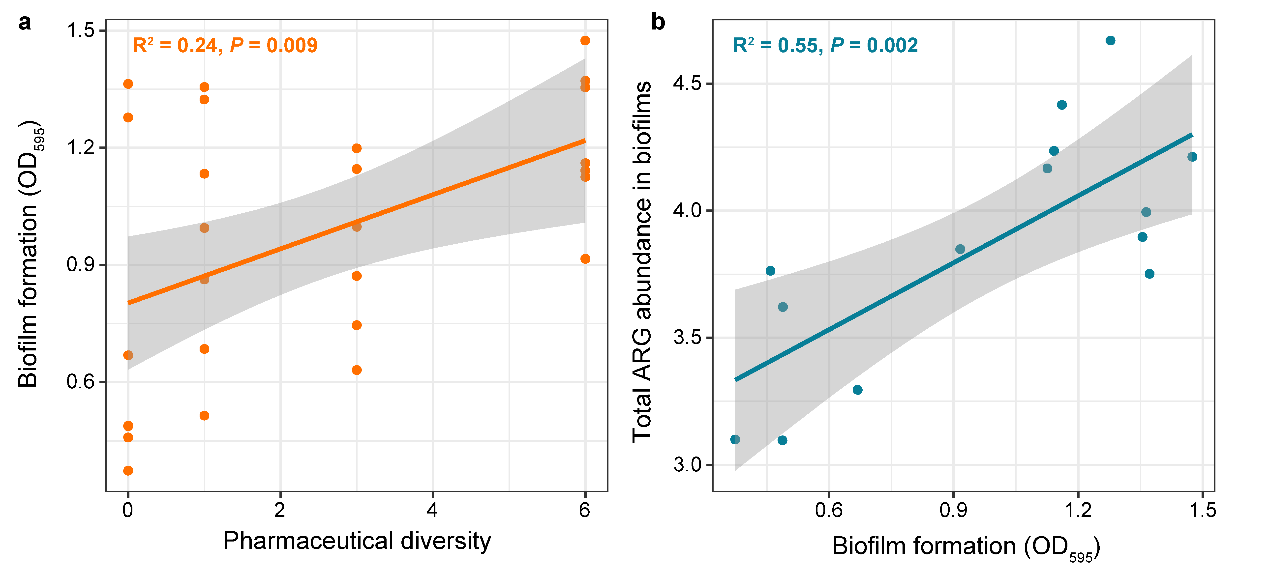
Fig. S4. Correlations between biofilm biomass and pharmaceutical diversity or ARG abundance.

a, Correlation between biofilm biomass (OD_595)_ of culturable collembolan gut microbiomes and pharmaceutical diversity (*n* = 28).

b, Correlation between biofilm biomass (OD_595_) and total relative abundance of ARGs in biofilm under control and six-pharmaceutical mixture exposure conditions (*n* = 14).

Shaded bands indicate 95% confidence intervals derived from linear regression analysis.


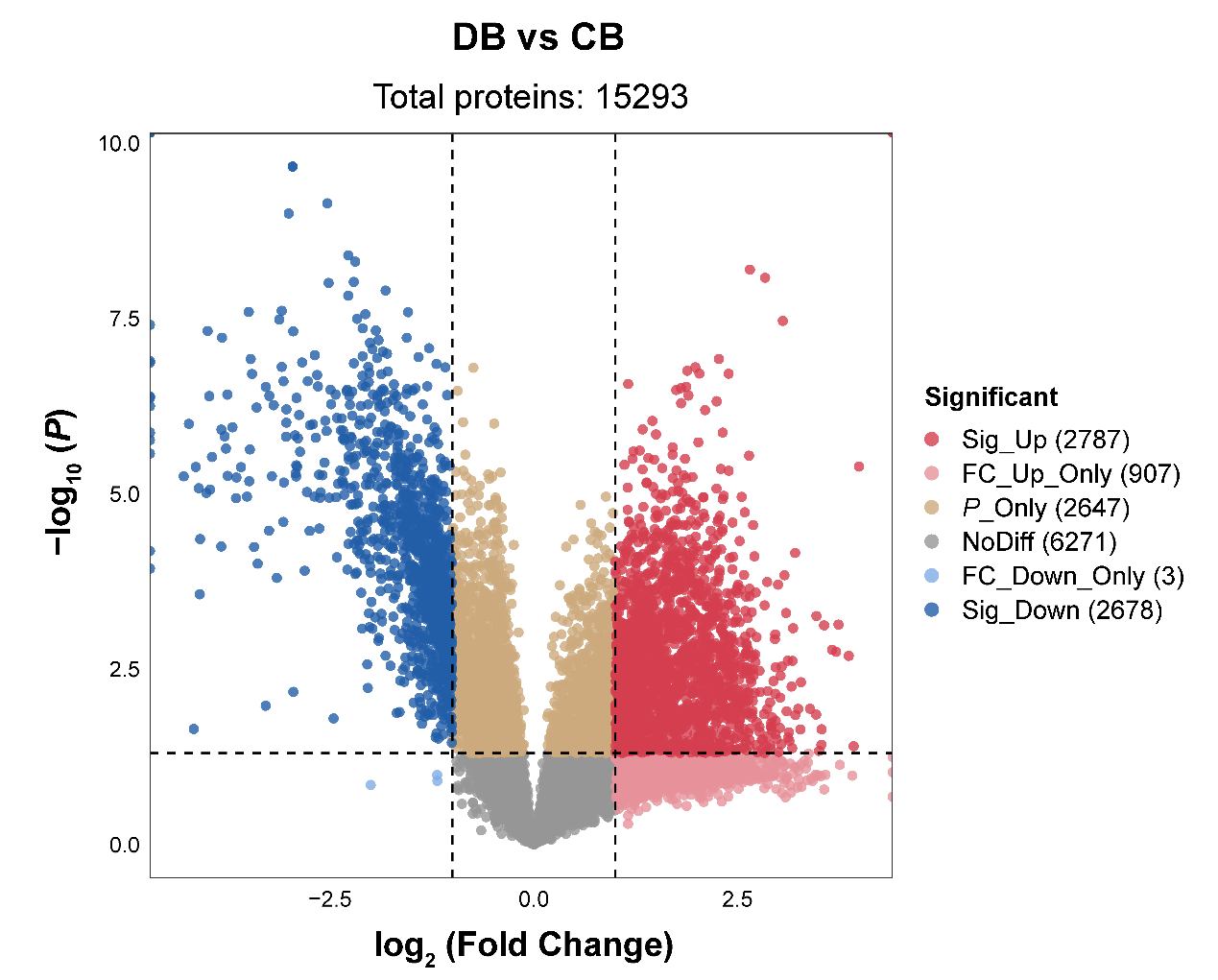


Fig. S5. Differentially expressed proteins between DB and CB groups.

Volcano plot showing log₂ fold change (x-axis) versus –log₁₀(P) value (y-axis) for 15,293 proteins. Proteins were categorized into seven groups based on statistical significance (*P* < 0.05) and fold change thresholds: significantly upregulated (Sig_Up, red), significantly downregulated (Sig_Down, blue), fold change only (FC_Up_Only and FC_Down_Only, light red and light blue), *P*-value only (*P*_Only, tan), no difference (NoDiff, grey). Thresholds for significance (horizontal dashed line) and fold change (vertical dashed lines at ±1.5 log₂) are indicated. Numbers in parentheses denote protein counts per category.


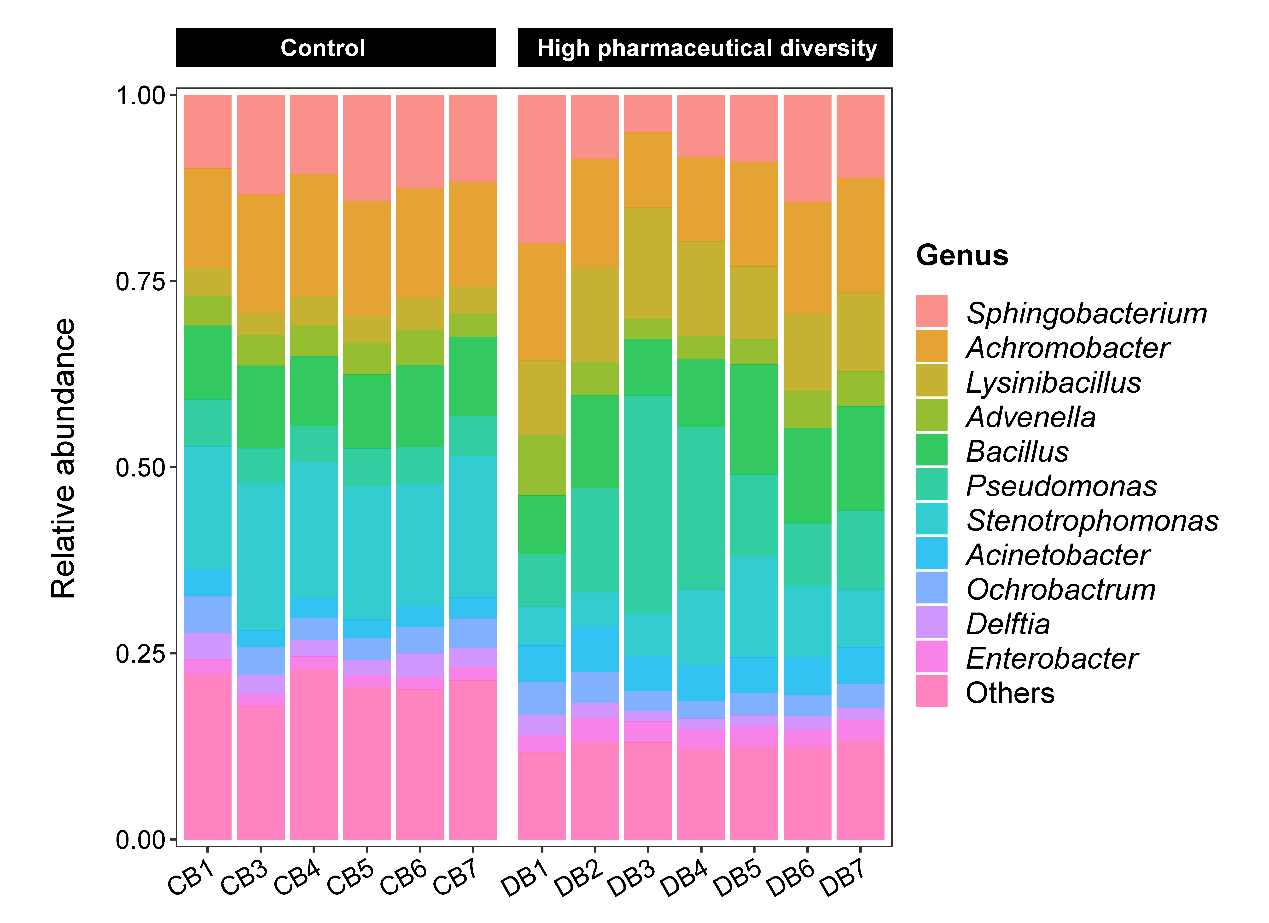


Fig. S6. Taxonomic annotation of biofilm proteomes under control and high pharmaceutical diversity treatments.

Genus-level annotation of identified proteins was performed to determine the major metabolically active bacteria in biofilm samples under control (non-drugs) and high pharmaceutical diversity (6 drug) exposure conditions.


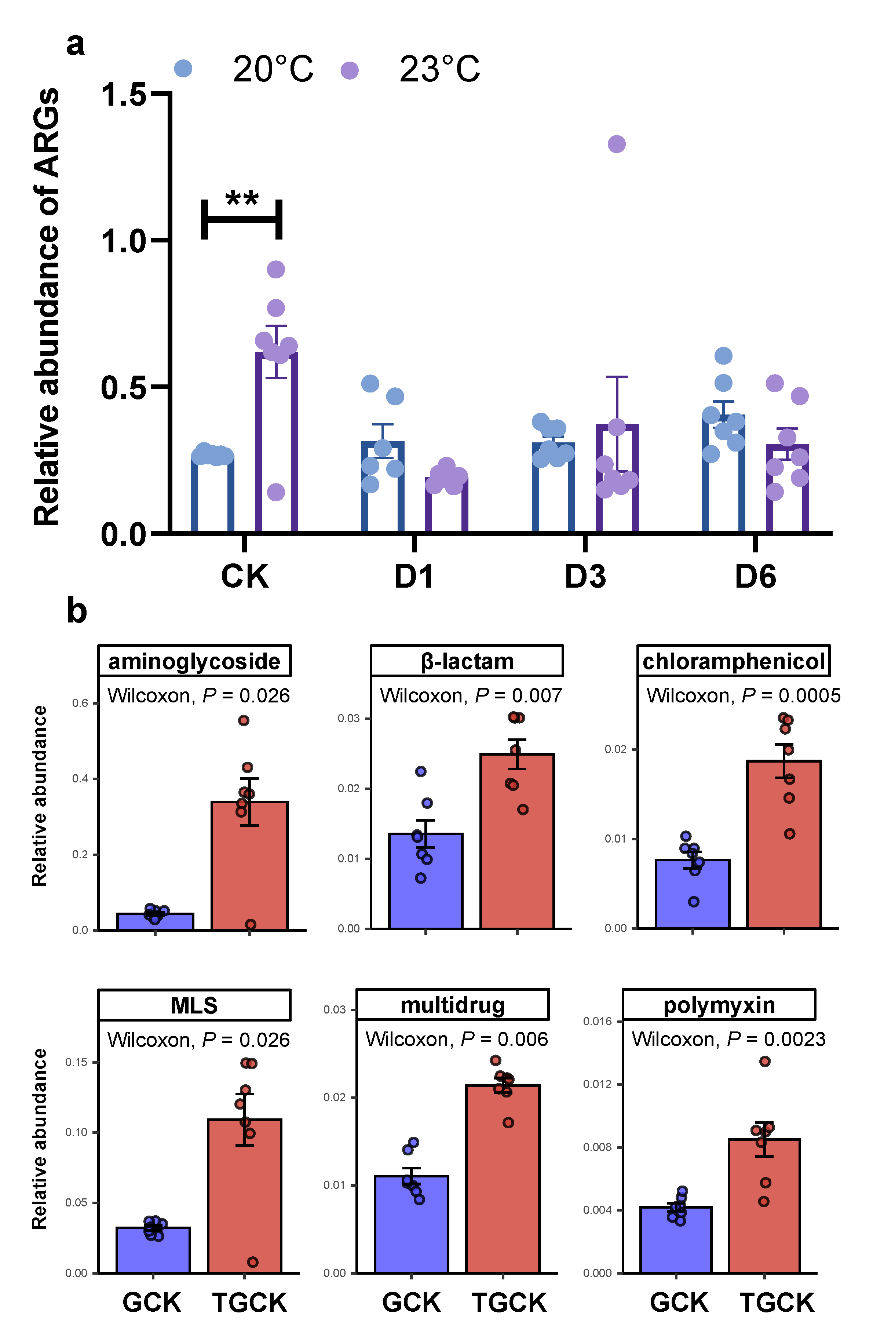


Fig. S7. Effects of warming and pharmaceutical diversity on ARGs in collembolan guts.

a, Relative abundance of total ARGs across treatments at 20 °C and 23 °C. A significant increase in ARG abundance was observed in the control group under warming. (CK: control; D1, D3, D6: 1, 3, and 6 drugs, respectively; *n* = 7 per group, except D1 at 20 °C where *n* = 6 due to one sample failing amplification).

b, Relative abundance of ARG types in GCK (20 °C) and TGCK (23 °C) groups. Error bars represent mean ± standard error (SE).


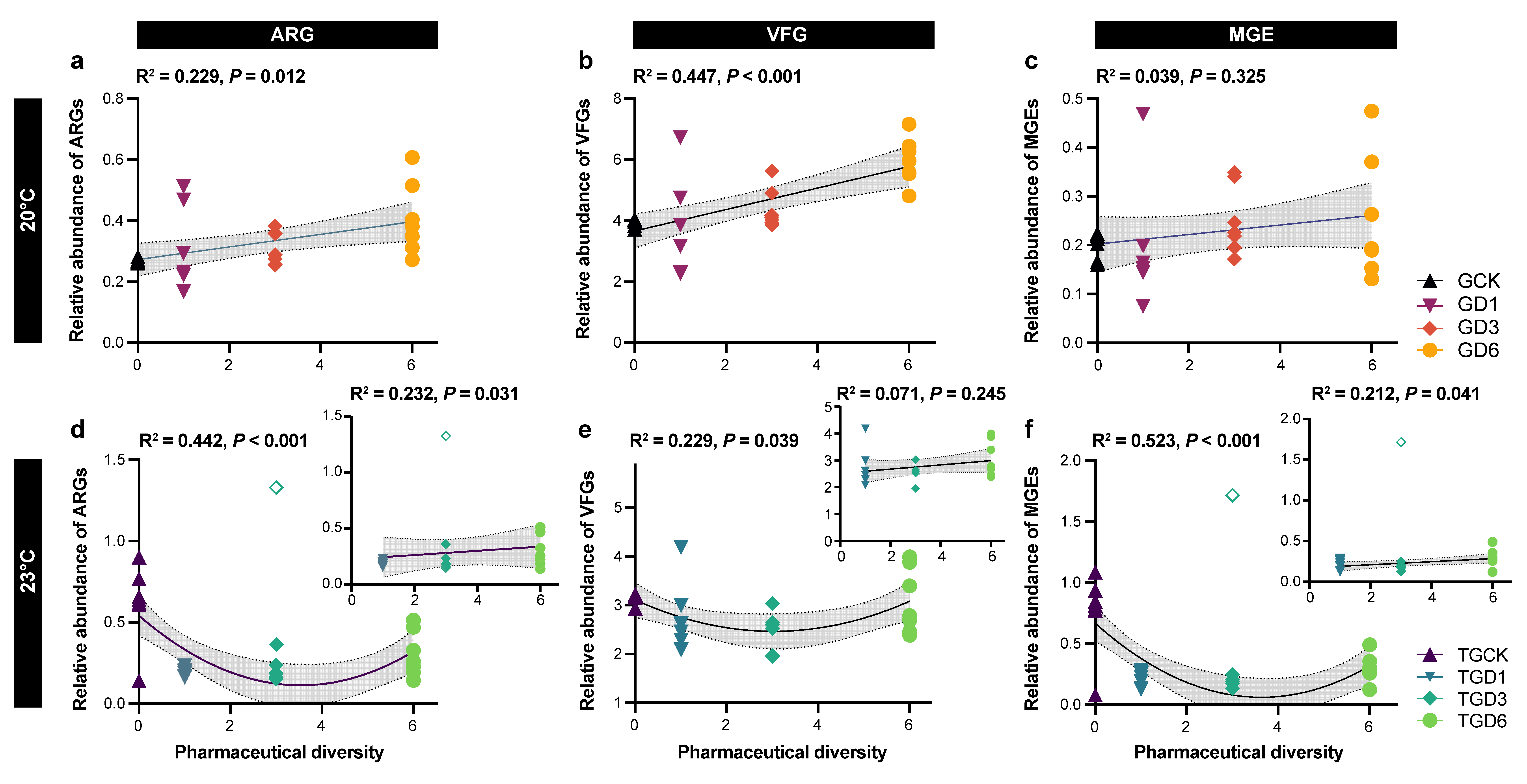


**Figure S8. Temperature influences the relationship between pharmaceutical diversity and ARG proliferation in collembolan gut.**

a, At 20 °C: linear regression between pharmaceutical diversity and total abundance of ARGs, VFGs, and MGEs in gut microbiomes (GCK: control; GD1, GD3, GD6: 1, 3, and 6 drugs, respectively; *n* = 7 per group, except GD1 where *n* = 6 due to one sample failing amplification).

b, At 23 °C: linear regression between pharmaceutical diversity and total abundance of ARGs, VFGs, and MGEs in gut microbiomes (TGCK: control; TGD1, TGD3, TGD6: 1, 3, and 6 drugs, respectively; *n* = 7 per group).

Shaded areas represent 95% confidence intervals from linear regression. The unit of genes is copies per cell.


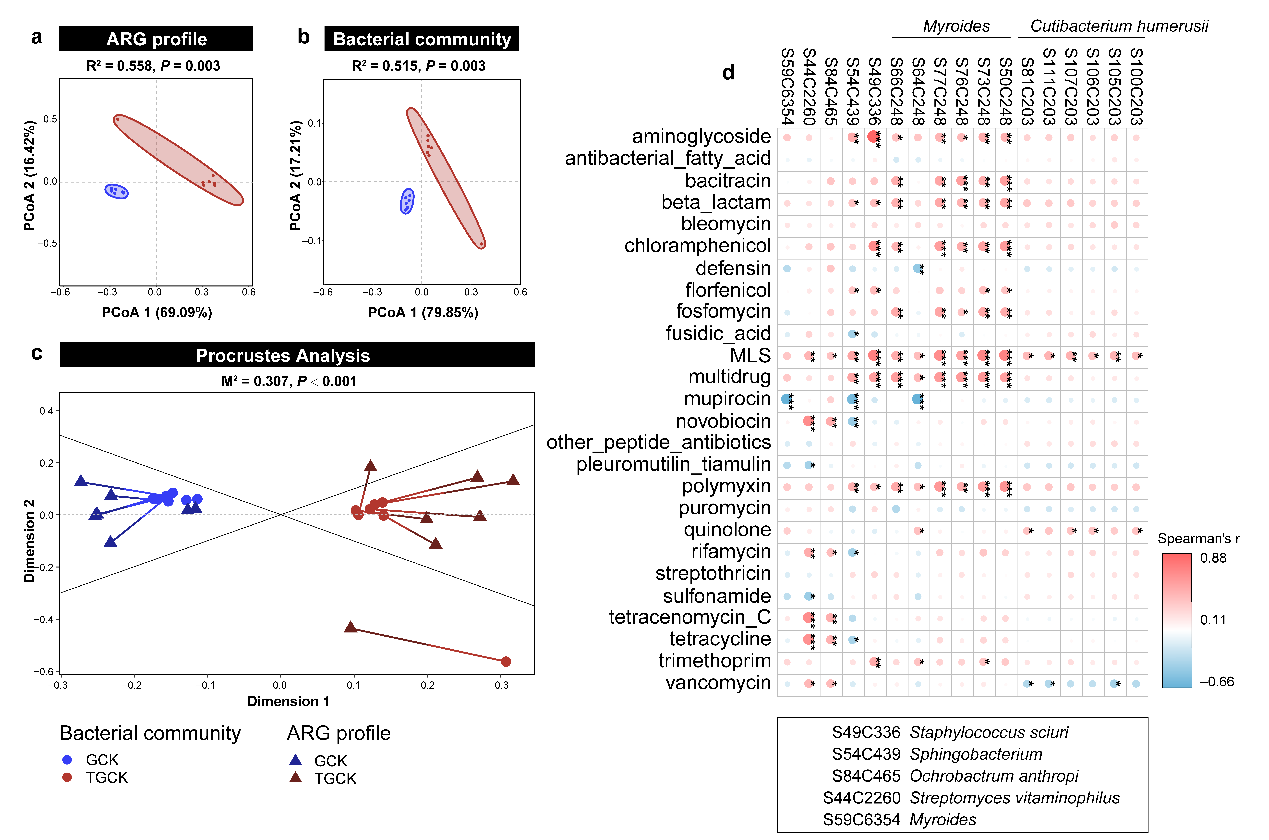


Fig. S9. Relationships between ARG profiles and bacterial community structure in collembolan guts under warming conditions.

a, Principal Coordinate Analysis (PCoA) based on Bray–Curtis distances of ARG profiles across treatments at 20 °C and 23 °C.

b, PCoA based on Bray–Curtis distances of bacterial community across treatments at 20 °C and 23 °C.

c, Procrustes analysis linking bacterial communities and ARG profiles, showing significant congruence.

d, Heatmap showing Spearman correlations between ARG types and dominant bacterial MAGs. Red indicates positive correlation and blue indicates negative correlation, with intensity reflecting strength. Significance among treatments is denoted as * *P* < 0.05, ** *P* < 0.01, *** *P* < 0.001.


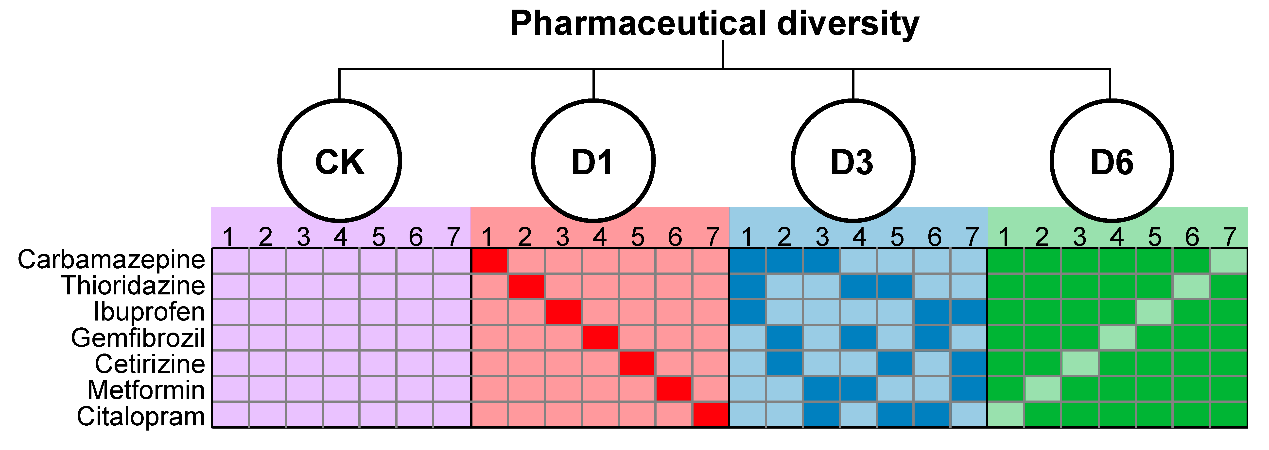


Fig. S10. Schematic overview of the microcosm experimental design.

The diagram illustrates the experimental setup used to investigate the effects of pharmaceutical diversity. Four levels of pharmaceutical diversity were established, including 0 (control, CK), 1 (D1), 3 (D3), and 6 (D6) different drug types
